# ClpB affects biofilm formation in methicillin-resistant *Staphylococcus aureus*

**DOI:** 10.3389/fmicb.2025.1723924

**Published:** 2025-12-04

**Authors:** Miao Yang, Shuang Wang, Qianwei Qu, Hai Yang, Xin Liu, Wei Peng, Yonghui Zhou

**Affiliations:** 1 School of Basic Medicine, Guizhou University of Traditional Chinese Medicine, Guiyang, Guizhou, China; 2 College of Veterinary Medicine, Northeast Agricultural University, Harbin, Heilongjiang, China; 3 Department of Pathology, The First Affiliated Hospital of Guizhou University of Traditional Chinese Medicine, Guiyang, Guizhou, China

**Keywords:** ClpB, MRSA, biofilm, skin infection, extracellular matrix

## Abstract

**Introduction:**

This study aims to explore the effects of the molecular chaperone ClpB on the biofilm formation and pathogenicity of methicillin-resistant *Staphylococcus aureus* (MRSA).

**Methods:**

The biological membrane formation was evaluated by constructing a *clpB* knockout strain (Δ*clpB*) and a complemented strain (CΔ*clpB*) of USA300 MRSA, followed by crystal violet staining, scanning electron microscopy, confocal laser scanning microscopy, and quantitative analysis of extracellular matrix components. A mouse skin infection model was subsequently employed to assess wound healing, histopathological changes, and the expression levels of inflammatory factors.

**Results:**

The results showed that compared with the wild strain (WT), the biomass of Δ*clpB* biofilm was significantly reduced (*p* < 0.0001), the structure was damaged and the production of extracellular matrix (eDNA, polysaccharides, proteins) decreased. CΔ*clpB* then returned to the WT level. In the *in vivo* experiments, the Δ*clpB* infection group had faster wound healing, reduced tissue damage, and decreased expressions of TNF-*α* and IL-6 at both protein and mRNA levels.

**Conclusion:**

ClpB promotes the formation of MRSA biofilms by regulating extracellular matrix synthesis and host inflammatory responses and is a potential target for anti-biofilm therapy.

## 1 Introduction

Methicillin-resistant *Staphylococcus aureus* (MRSA) has emerged as a formidable pathogen, causing significant morbidity and mortality by evading host immune defenses through various mechanisms. Initially identified in the 1960s, MRSA has since developed multiple strategies for antimicrobial resistance and immune system evasion, enabling it to induce severe diseases, including those characterized by biofilm formation (Kaushik et al., 2024; Jing et al., 2022). With a diverse repertoire of evasion tactics targeting host defenses, MRSA has become a pervasive pathogen responsible for a spectrum of infections, ranging from persistent skin and soft tissue infections (SSTIs) to more entrenched conditions such as bone and joint infections and endocarditis (Jiang et al., 2023; Nigo et al., 2024; Chen et al., 2025). The limited treatment options for MRSA are compounded by its biofilm-forming capability, a key factor in its pathogenicity and resistance. Research indicates that approximately 60% of *in vivo* infections are attributed to bacteria encased in biofilms, perpetuating the disease process (Kaushik et al., 2024). Biofilms are intricate structures formed by bacteria adhering to surfaces, comprising various molecules such as bacterial extracellular matrix components, proteins, polysaccharides and DNA. These biofilms shield the strain from host immune responses, enabling it to circumvent host defenses. Furthermore, biofilms enhance the strain’s capacity to adhere to host tissues, facilitating colonization and growth within the host, thereby heightening susceptibility to infection and exacerbating treatment challenges (Aboelnaga et al., 2024).

Biofilm formation is a multifaceted process governed by a network of genes, proteins and regulatory pathways. Previous research has identified specific genes and proteins linked to biofilm formation in MRSA strains (Shang et al., 2022; Miao et al., 2024). Notably, proteomic analysis of *S. aureus* exposed to clemastine revealed significant alterations in biofilm-related proteins (such as stress response regulators ClpB and GroS, ATP-binding proteins, and urease metabolism), toxic-related proteins (including SspA, superantigen, and VWbp) and methicillin-resistance-related proteins (like those involved in glutamine metabolism) (Shang et al., 2022). Our prior investigation demonstrated that ClpB was the most markedly down-regulated protein among the differentially expressed proteins in MRSA USA300 treated with tannic acid, as detected through proteomic analysis (Miao et al., 2024). Building upon these findings, we have undertaken a detailed examination of the association between ClpB and MRSA biofilm formation in present study.

The ClpB, a member of the Hsp100 family, functions as a molecular chaperone in bacteria, aiding in proper protein folding and assembly during stress conditions (Yang et al., 2024). Pavla Pavlik highlighted in a study the multifunctionality of ClpB, emphasizing its high conservation across bacterial species and its involvement in various stress responses and toxicity mechanisms (Pavlik and Spidlova, 2022). Notably, ClpB has been associated with biofilm formation in *Porphyromonas gingivalis* and *Bacillus amylolytica* (Wiktorczyk-Kapischke et al., 2023; Peeran et al., 2024). However, the impact of ClpB on MRSA biofilm formation requires additional investigation. In *S. aureus* infection, the pathogen’s virulence is attributed to the production of toxic factors such as the hla toxin, which binds to target cell membranes, leading to cellular damage (Jing et al., 2022). Studies have shown that a *P. gingivalis clpB* mutant exhibited decreased invasiveness and virulence in a mouse infection model (Kędzierska-Mieszkowska and Zolkiewski, 2021). Similarly, *clpB* mutants in *Listeria monocytogenes*, *Salmonella typhi*, and *Mycobacterium tuberculosis* displayed significantly reduced virulence in infection models (Kędzierska-Mieszkowska and Zolkiewski, 2021). Particularly in *Mycobacterium tuberculosis*, ClpB is essential for survival under stress and contributes to regulating its toxicity (Kędzierska-Mieszkowska and Zolkiewski, 2021). Collectively, these findings underscore the critical role of ClpB in bacterial pathogens’ invasion of hosts, rapid adaptation, survival, replication, and evasion of host defenses. However, further research is needed to confirm the impact of ClpB on MRSA biofilm.

In this investigation, the ClpB protein in MRSA strains was identified as a protein associated with biofilm formation. To elucidate the role of ClpB in MRSA biofilm formation, we produced mutant strains lacking the *clpB* gene (Δ*clpB*), complemented strains (CΔ*clpB*), and empty plasmids (Δ*clpB-*pCM), and conducted a comprehensive set of experiments to characterize them. These findings lay the groundwork for further exploration and identification of potential protein targets for inhibiting MRSA biofilm formation.

## 2 Materials and methods

### 2.1 Strain source and culture conditions

The *S. aureus* strains USA300, RN4220, and plasmid pKOR1 utilized in this study were generously provided by Professor Yanhua Li from Northeast Agricultural University and maintained in our laboratory.

The strains stored at −80 °C were thawed at room temperature and inoculated into three regions of Trypticase Soy Agar (TSA, HB4114, Qingdao, China) plates in a biosafety cabinet. The plate was then incubated upside down at 37 °C for 20–24 h. Single colonies of suitable size were chosen and transferred into 5 mL of Tryptic Soy Broth (TSB, HB4114, Qingdao, China) liquid medium. The culture was maintained at 37 °C on a constant temperature shaking table until reaching an optical density at 600 nm (OD600) of 1–1.5.

### 2.2 Experimental method

#### 2.2.1 Construction and identification of MRSA USA300 Δ*clpB*, CΔ*clpB* and Δ*clpB-*pCM

##### 2.2.1.1 Primer design

In this study, PCR primers were designed using Snapgene software based on the *clpB* gene sequence and its genomic context within the complete USA300 genome. The upstream and downstream homologous arms used for amplification were 994 bp and 995 bp in length, respectively. To facilitate seamless cloning with the linearized vector, the 3′ end of the upstream homologous arm primer and the 5′ end of the downstream homologous arm primer were designed to include overlapping regions of 15–20 bp. Furthermore, to enable fusion of the homologous arm fragments with the knockout vector pKOR1, overlapping sequences of 15–20 bp were incorporated between the 5′ end of the upstream homologous arm primer and the 3′ end of the pKOR1 primer, as well as between the 3′ end of the downstream homologous arm primer and the 5′ end of the pKOR1 primer. The primers used for amplification of the upstream homologous arm were *clpB*-19-F and *clpB*-up-R, while *clpB*-down-F and *clpB*-19-R were used for the downstream homologous arm. Primers pKOR1-*clpB*-F and pKOR1-*clpB*-R were designed based on the pKOR1 vector sequence to construct the vector backbone. Primer pKOR1-JD-R was employed for sequencing the knockout vector, whereas *clpB*-up-F and *clpB*-down-R were used for post-transformation verification. Additionally, *clpB*-ter-F and *clpB*-ter-R were utilized to amplify the full-length *clpB* gene for knockout confirmation. The primer pair *clpB*-JD-F and *clpB*-JD-R was used to amplify a region spanning 203 bp upstream to 280 bp downstream of the *clpB* gene to further validate the knockout efficiency. The primer sequences employed in the construction of the USA300 **Δ***clpB* strains are summarized in Table 1, and those used for the CΔ*clpB* strains are listed in Table 2. All primers were synthesized by Shanghai Shenggong Biotechnology Co., LTD. For detailed information regarding the USA300 **Δ***clpB* and CΔ*clpB* strains, refer to Supplementary material 1.

**Table 1:** Primers used in this experiment.

Primer name	Primer sequence
*clpB*-19-F	TGTAAAACGACGGCCAGTGCAGAGCTATCATATCGCTTTAT
*clpB*-19-R	CTATGACCATGATTACGAAGAGGTAAGTTCATGAATTTAG
*clpB*-up-R	ACATTCTTGGTTCAATTATAATTTCACCTCTAATTTGTAG
*clpB*-down-F	AATTAGAGGTGAAATTATAATTGAACCAAGAATGTGATGA
pKOR1-*clpB*-R	ATATGATAGCTCTGCGGTACCGGTTCCGAGGCTCAACGTC
pKOR1-*clpB*-F	TCATGAACTTACCTCTTGCGTTGCGCTCACTGCCCGCTTT
*clpB*-pKOR1-F	GCCTCGGAACCGGTACCGCAGAGCTATCATATCGCTTTAT
*clpB*-pKOR1-R	GGCAGTGAGCGCAACGCAAGAGGTAAGTTCATGAATTTAG
*clpB*-JD-F	GACGGTGTACATTTAGAGTACAA
*clpB*-JD-R	TAATAAGTGATAACCATCCAGAA
*clpB*-ter-F	TGAAGACAAACTAAACACGTA
*clpB*-ter-R	TCTGTTTGGATCTTTAATACC

**Table 2:** Primers used in this experiment.

Primer name	Primer sequence
pCM-his-F	CACCACCACCACCACCACTAAGAATTCGTAATCATGTCAT
pCM-*clpB*-R	AGACTTATTAAACATATGGACACAGTGATTGTATTTCTGG
*clpB*-pCM-F	AAATACAATCACTGTGTCCATATGTTTAATAAGTCTAGTT
*clpB*-his-R	GTGGTGGTGGTGGTGGTGTTCATGAATTTTTTCAACATTAA
*clpB*-CX-F	TTCGGCATTAGAGCGTCGTTTCC
M13-R	AGCGGATAACAATTTCACACAGG

##### 2.2.1.2 MRSA USA300 DNA extraction

Individual colonies of suitable size were chosen and inoculated into 5 mL of TSB liquid medium. The cultures were then incubated at 37 °C on a shaking table until reaching an optical density at 600 nm of 1–1.5. Subsequently, 2 mL of the bacterial suspension was centrifuged at 5000 × g for 10 min, and the supernatant was discarded to harvest the bacterial cells. The DNA extraction of *S. aureus* was carried out following the protocol provided by the Takara Bacterial Genomic DNA Small Amount Extraction Kit (Baori Doctor Biological Technology Co., LTD., Beijing, China).

##### 2.2.1.3 Preparation of Δ*clpB* and CΔ*clpB* strains

The primers *clpB*-19-F, *clpB*-up-R, *clpB*-down-F, and *clpB*-19-R were utilized to set up the reaction system with extracted USA300 DNA as the template (refer to Supplementary material 1 for detailed procedures). PCR was employed to amplify the upstream and downstream homologous arms, followed by gel recovery of the amplified fragments. Subsequently, the amplified *clpB* up and *clpB* down fragments were seamlessly cloned into the respective homologous arms. The resulting constructs were then placed on ice for 30 min, chemically transformed into DH5α competent cells, plated on Ampicillin-resistant plates (Amp, CAS:69-52-3, Beijing Solarbio Technology Co., LTD., China), and incubated at 37 °C for white colony selection and sequencing. Using the *clpB*-ud-puc19 plasmid as a template, the carrier framework was generated using primers *clpB*-pKOR1-F and *clpB*-pKOR1-R, with the primer pKOR1-*clpB*-F/pKOR1-*clpB*-R serving as the carrier. The fusion fragments of the upstream and downstream homologous arms and the prepared pKOR1 backbone products were seamlessly cloned, chilled on ice for 30 min, chemically transformed into DH5α competent cells, and plated on Ampicillin-resistant plates for screening. The pKOR1-*clpB* ud plasmid, containing the sequences of the upstream and downstream homologous arms of *clpB*, was introduced into RN4220 recipient cells via electrotransformation at 2300KV and plated on TSA plates supplemented with Chloramphenicol (CAS:56-75-7, Beijing Solarbio Technology Co., LTD., China) (Cm 5 μg/mL) for cultivation at 30 °C. Subsequently, RN4220-*clpB*-pKOR1 phage was prepared, and USA300 was transformed using the phage transduction method, followed by cultivation at 30 °C on TSA plates containing Cm 10 μg/mL. Positive clones were inoculated in 5 mL TSB (Cm 10 μg/mL) liquid medium at 30 °C, transferred to fresh 5 mL TSB (Cm 10 μg/mL) the next day for overnight incubation at 43 °C, and then plated on TSA (Cm 7.5 μg/mL) at 43 °C. Selected clones were further transferred to fresh 5 mL TSB (Cm 5 μg/mL) for overnight culture at 43 °C, plated on TSA at 30 °C, and the resulting clones were transferred to 5 mL TSB for culture at 30 °C. The bacterial solution was diluted and plated on Anhydrotetracycline Hydrochloride (ATC 1 μg/mL) (CAS: 13803-65-1, Shanghai Yuanye Biotechnology Co., Ltd., China) plates, where the clones were able to grow. Colonies were then streaked on TSA plates and TSA (Cm 10 μg/mL) plates, and short clones from the chloramphenicol plate were selected for identification using the primers *clpB-*JD-F/*clpB*-JD-R and *clpB*-ter-F/*clpB*-ter-R. The complemented strain can be found in Supplementary material 1. At the same time, we also constructed an empty plasmid vector (USA300 Δ*clpB-*pCM). To verify whether the CΔ*clpB* strain was successful, the construction process was the same as that of the CΔ*clpB* strain.

#### 2.2.2 Detection of growth curves of MRSA USA300 Δ*clpB*, CΔ*clpB*, Δ*clpB-*pCM and wild-type strains

Single colonies of the USA300 Δ*clpB,* CΔ*clpB,* Δ*clpB-*pCM and wild-type strains were selected from TSA plates under aseptic conditions and inoculated into sterile TSB liquid medium. Following incubation at 37 °C with shaking at 200 rpm until reaching the logarithmic growth phase, the bacterial solution was diluted to 1×10^6 as the initial bacterial solution. Subsequently, 200 μL of the diluted bacterial solution was dispensed into a 96-well tissue culture plate, with sterile TSB culture solution designated as the blank control group. The cultures were statically incubated in a constant temperature incubator at 37 °C. The OD 600 _nm_ value was monitored at 1, 2, 3, 4, 5, 6, 7, and up to 14 h post-culture, with each time point being replicated thrice. The growth curve was generated by plotting the culture duration on the x-axis and the corresponding OD 600 _nm_ values on the y-axis. All experiments were conducted in triplicate to ensure reproducibility and reliability.

#### 2.2.3 Detection of biofilm-forming ability of MRSA USA300 Δ*clpB*, CΔ*clpB*, Δ*clpB*-pCM and wild-type strains

##### 2.2.3.1 Crystal violet stain detection

Single colonies of the USA300 Δ*clpB,* CΔ*clpB,* Δ*clpB-*pCM and wild-type strains were selected from TSA plates under aseptic conditions and inoculated into sterile TSB liquid medium. Following incubation at 37 °C with shaking at 200 rpm until reaching the logarithmic growth phase, the bacterial suspension was diluted to a concentration of 1×10^6 colony-forming units per milliliter (CFU/mL). Subsequently, 200 μL of the diluted bacterial suspension was added to each well of a 96-well tissue culture plate, with 6 replicates per strain. Sterile TSB medium served as the blank control, and the plates were statically incubated at 37 °C. After 24 h, the plates were processed to assess biofilm formation. The bacterial suspension was discarded, and wells were washed with 200 μL of sterile PBS until no floating bacteria were visible. Next, 200 μL of methanol was added to each well for fixation for 15 min. Following removal of methanol and rapid air-drying, 200 μL of 0.1% crystal violet staining solution was added to each well for 5 min. Excess dye was rinsed off with deionized water, and the plates were air-dried. Biofilm-bound crystal violet dye was solubilized by adding 200 μL of glacial acetic acid and incubating for 30 min. Optical density (OD) values were measured at 570 nm using a Thermo Fisher Multiskan FC spectrophotometer, with each experiment conducted in triplicate. The reagents used in this experiment, such as crystal violet, methanol, ethanol, glacial acetic acid, absolute ethanol, glutaraldehyde, tert-butyl alcohol, phosphate- buffered saline (PBS), and other reagents were purchased from Tianjin KOMIO Chemical Reagents Company, Ltd. (Tianjin, China).

##### 2.2.3.2 Detection by scanning electron microscopy

After inducing expression of the USA300 Δ*clpB,* CΔ*clpB* and wild-type strains, the bacterial solution was diluted to 1×10^6 colony-forming units per milliliter (CFU/mL) in Tryptic Soy Broth (TSB) medium. Subsequently, 2 mL of the bacterial solution was inoculated into a 6-well tissue culture plate with a sterile ground glass coverslip and then incubated at a constant temperature of 37 °C. Following a 24 h incubation period, the coverslips were retrieved, and the adherent bacteria were gently rinsed with sterile phosphate-buffered saline (PBS) solution. The coverslips were then immersed in 5% glutaraldehyde and fixed overnight at 4 °C in the dark. Subsequently, the coverslips were sequentially rinsed twice with PBS for 10 min each, followed by dehydration in 50, 70, and 90% ethanol for 15 min each, and finally in 100% ethanol for 15 min. This was followed by a treatment with a 1:1 mixture of 100% ethanol and tert-butanol, and pure tert-butanol, each for 15 min. The samples were then subjected to freeze-drying for 4 h, after which a 150 Å thick metal film was sputtered onto the sample surface under vacuum conditions. The morphology of the biofilm was examined using a scanning electron microscope (HITACHI, model SU8010, Japan) to elucidate the role of ClpB in methicillin-resistant *S. aureus* (MRSA) biofilm formation. The experiment was conducted in triplicate for statistical robustness.

##### 2.2.3.3 Laser confocal microscopy

After culturing the USA300 Δ*clpB,* CΔ*clpB* and wild-type strains, the bacterial solution was diluted to 1×10^6 colony-forming units per milliliter (CFU/mL) in Tryptic Soy Broth (TSB) medium. Subsequently, 2 mL of the bacterial solution was transferred to a confocal culture dish and incubated at a constant temperature of 37 °C. After 24 h, the petri dish was removed, the bacterial solution was aspirated, and the dish was rinsed thrice with sterile PBS (2 mL) before being fixed with 2 mL of 2.5% glutaraldehyde for 1.5 h. Following fixation, the dish was washed thrice with PBS buffer. Staining was carried out using SYTO 9 (Item number: PS1384-40 T, Shenzhen Zetao Biotechnology Co., LTD.) by adding 20 μL of the dye solution to the culture dish and allowing it to incubate for 15 min. Subsequently, the dish was rinsed with Milli-Q water and carefully dried to eliminate excess water. The dish was then mounted on the instrument, and a sealing oil (BacLight) was applied. The formation of fluorescent biofilms was visualized using a 710 nm confocal laser scanning microscope (CLSM) (Leica, model TCS SP8, Germany), and images were captured for verification.

#### 2.2.4 Detection of biofilm matrix of MRSA USA300 Δ*clpB*, CΔ*clpB* and wild-type strains

Following Siddhi Desai’s protocol (Desai et al., 2019), the Δ*clpB*, CΔ*clpB* and wild-type strains were inoculated into TSB liquid medium and incubated at 37 °C with shaking at 200 rpm for 16 h. The bacterial suspension was then adjusted to a concentration of 1 × 10^6 CFU/mL. Subsequently, 1 mL of the bacterial suspension was transferred into each well of a 24-well tissue culture plate containing 3 mL of TSB medium supplemented with 1% glucose. Six biological replicates were established for each group. The plates were incubated statically at 37 °C for 72 h. After the incubation period, the TSB medium was carefully aspirated from the wells, leaving behind the biofilm formed at the bottom of the wells. The biofilm was resuspended in 3 mL of 0.8% physiological saline, and 1.5 mL of this suspension was collected for extracellular polysaccharide quantification (Sample A). To the remaining 1.5 mL of the physiological saline suspension, sodium dodecyl sulfate (SDS) was added to achieve a final concentration of 0.01%, followed by incubation at room temperature with shaking at 150 rpm for 4 h. The resulting solution was centrifuged at 5000 × g for 5 min to remove cellular debris, and the supernatant was filtered through a 0.22 μm cellulose acetate filter membrane. The filtrate was subsequently used for the quantification of extracellular DNA and proteins (Sample B). Extracellular protein quantification was performed using the Bradford assay (Product Code: PC0010, Beijing Solabao Technology Co., Ltd., China). Bovine serum albumin (Product Code: PC0010, Beijing Solabao Technology Co., Ltd., China) served as the standard for calibration. A 100 μL aliquot of Sample B was mixed with 1 mL of Bradford reagent. The mixture was incubated at room temperature in the dark for 10 min. Absorbance was measured at 595 nm using a microplate reader (three technical replicates). The protein concentration (mg/mL) was determined based on the regression equation derived from the standard curve. Polysaccharide determination: The polysaccharide content was quantified using the phenol-sulfuric acid assay method with a commercial kit (JL-T0827, Shanghai Jianglai Biotechnology Co., Ltd., China). Absorbance was measured at 488 nm, and a glucose solution (AnalaR NORMAPUR^®^) served as the standard. Specifically, 200 μL of Sample A was mixed with 100 μL of 5% phenol solution, followed by the slow addition of 500 μL of concentrated sulfuric acid while maintaining an ice bath to prevent overheating. The mixture was incubated at room temperature for 30 min. Subsequently, absorbance was measured at 488 nm, and the polysaccharide content was calculated based on the glucose standard curve. eDNA determination: For eDNA extraction, 1 mL of Sample B was combined with an equal volume of phenol: chloroform: isopentanol (25:24:1), vortexed thoroughly, and centrifuged at 12,000 × g for 10 min at 4 °C. The supernatant was carefully transferred to a new tube, and 1/10 volume of 3 M sodium acetate (pH 5.2) and twice the volume of pre-cooled anhydrous ethanol were added to precipitate the DNA overnight at −20 °C. The sample was then centrifuged at 16,000 × g for 30 min at 4 °C, and the pellet was washed with 70% ethanol before being centrifuged again at 16,000 × g for 2 min. After drying at room temperature for 10 min, the DNA was dissolved in 20 μL of DEPC-treated water. The DNA concentration was determined using a Nanodrop™ spectrophotometer (Thermo Scientific™, United States) by measuring absorbance at 260 nm.

#### 2.2.5 *In vivo* experiment

All animal experiments conducted in this study were approved by the Ethics Committee of Guizhou University of Traditional Chinese Medicine (Approval Number: 20250312004). A total of fifty specific pathogen-free (SPF)-grade Kunming mice (male, 5–6 weeks of age, weighing 20–22 g) were procured from Henan Skobes Biotechnology Co., LTD. (License Number: SCXK(YU)2020-0005). Following a 7-day acclimatization period in an SPF-grade animal facility to minimize stress responses, the animals were randomly assigned to four experimental groups: Group A, Group B, Group C, Group D and Group E (*n* = 10 per group). Wound assessment, histological analysis, and ELISA measurements were performed by investigators blinded to the group allocations.

Single colonies of the WT strain and the Δ*clpB* strain were separately inoculated into TSB medium and incubated at 37 °C with shaking at 200 rpm for 16 h. The bacterial suspensions were subsequently adjusted to a concentration of 1×10^6 CFU/mL and held on standby for use. Prior to model establishment, dorsal fur was removed using an electric clipper and the skin was disinfected with iodophor. On the day of modeling, mice were anesthetized via intraperitoneal injection of Zoletil 50 (40 mg/kg, Tianjin Bailaiyuan Biotechnology Co., Ltd.), and a standardized burn injury was induced by applying a preheated metal plate (100 °C, 4 × 4 cm) to the dorsal skin for 10 s under non-contact pressure conditions.

The experimental group assignments were as follows: Group A (Blank Control Group): No burn injury or additional intervention. Group B (Burn Control Group): Wounds were injected with an equal volume of sterile phosphate-buffered saline (PBS). Group C (Wild-Type Strain Group): 100 μL of MRSA wild-type bacterial suspension (1×10^6 CFU/mL) was injected onto the wound surface. Group D (Δ*clpB* Strain Group): 100 μL of MRSA Δ*clpB* strain suspension (1×10^6 CFU/mL) was injected onto the wound surface. Group E (Vancomycin Treatment Group): 100 μL of MRSA wild-type bacterial suspension (1×10^6 CFU/mL) was injected onto the wound surface and applied 100 μL MIC (3.90625 μg/mL) vancomycin to the infected wound every 24 h. Group E was an additional positive control without affecting the main study. Following model induction, all animals received fluid resuscitation and were provided analgesia as required. Animals exhibiting weight loss exceeding 20%, inability to feed independently, or signs of severe systemic infection were humanely euthanized in accordance with ethical guidelines.

Wounds were monitored and photographed on days 1, 3, 5, 7, 9, and 14. At days 3, 5, and 7, three mice per group were randomly euthanized via cervical dislocation. The excised wound tissue was fixed in 4% paraformaldehyde, followed by hematoxylin and eosin staining for histopathological examination. The tissue was then weighed, homogenized in 1 mL sterile normal saline per gram of tissue on ice, and centrifuged at 3000 g for 10 min. The resulting supernatant was divided for colony counting and enzyme-linked immunosorbent assay (ELISA) analysis of tumor necrosis factor-*α* (TNF-α) and interleukin-6 (IL-6) levels using a commercial kit (Wuhan Enzyme Free Biotechnology Co., LTD.). Total RNA was extracted from the remaining tissue using RNAiso Plus and chloroform. Real-time quantitative reverse transcription polymerase chain reaction (RT-PCR) was employed to determine the transcription levels of TNF-α and IL-6. The primer sequences used were as follows: Mus GAPDH (Forward: GAGAGTGTTTCCTCGTCCCGTA, Reverse: CCTCACCCCATTTGATGTTAGT), Mus IL-6 (Forward: ACAACCACGGCCTTCCCTACT, Reverse: TTCTCATTTCCACGA TTTCCC), and Mus TNF-α (Forward: TGGAACTGGCAGAAG AGGCAC, Reverse: CCATAGAACTGATGAGAGGGA). The RT-PCR protocol included an initial denaturation step at 95 °C for 10 min, followed by 40 cycles of denaturation at 95 °C for 15 s, annealing and elongation at 60 °C for 60 s, and a final melting curve analysis.

### 2.3 Statistical analysis

Each trial was conducted in triplicate, and standard deviations (SD) were computed. Statistical analyses were carried out using Prism 10. software (GraphPad, San Diego, CA, United States) through one-way ANOVA and Student’s *t*-test. Statistical significance was set at *p* < 0.05.

## 3 Results

### 3.1 Construction of USA300 Δ*clpB*, CΔ*clpB*, and Δ*clpB-*pCM

**Figure 1:**
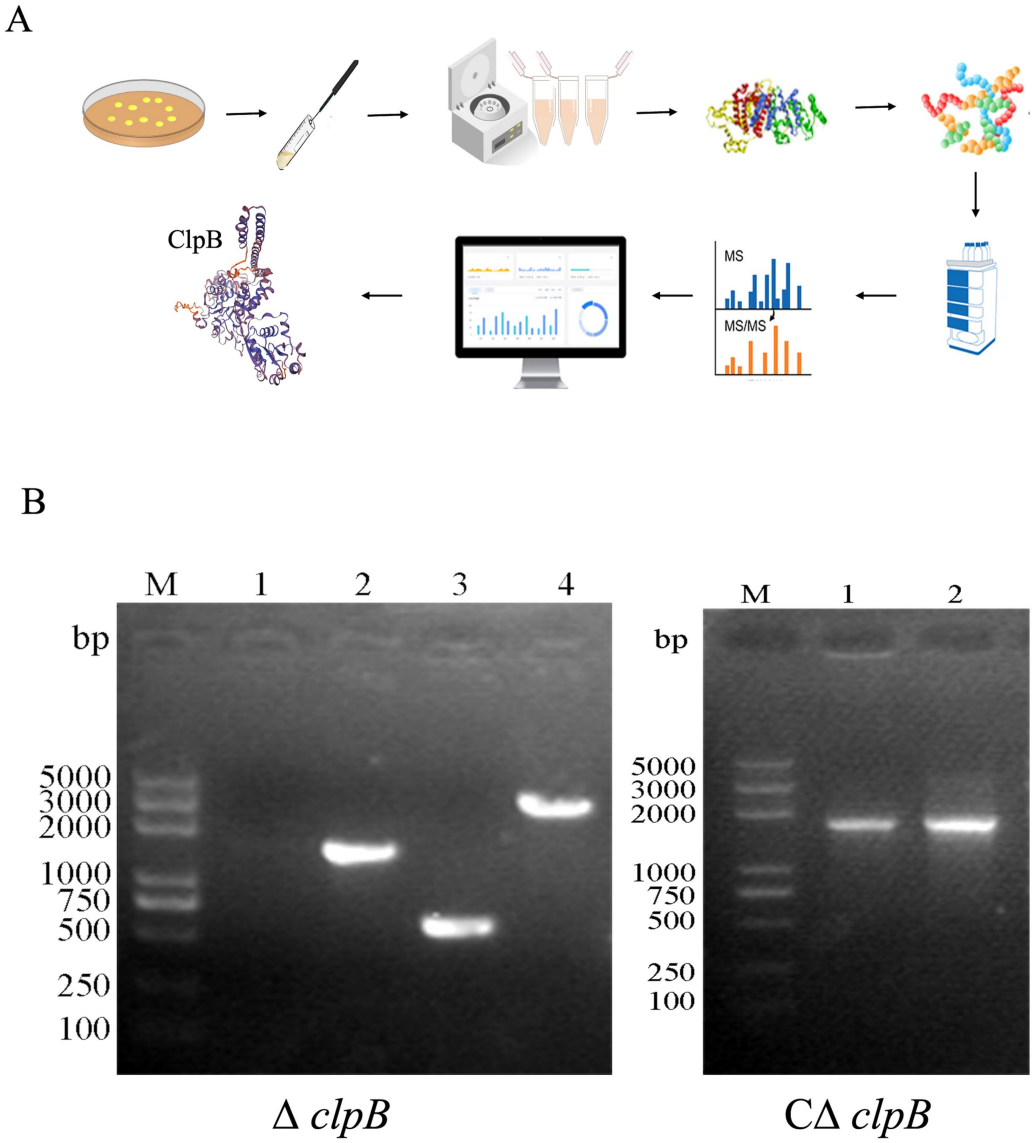
The discovery of ClpB as a potential target protein influencing MRSA biofilm and the construction of *clpB* gene knockout (Δ*clpB*) and complemented strains (CΔ*clpB*). **(A)** Schematic diagram of screening differential proteins; **(B)** Identification and amplification detection of Δ*clpB* and CΔ*clpB*.

In our previous study, we identified ClpB as the most significantly down-regulated protein impacting biofilms, as depicted in Figure 1A, using proteomics technology. Subsequently, we targeted ClpB for further investigation. After using homologous recombinant gene knockout technology, in the left figure of Figure 1B, No. 1 represents the *clpB*-ter-F/R amplification product of the USA300Δ*clpB* strain, while No. 2 corresponds to the *clpB*-ter-F/R amplification product of the USA300 wild-type strain. No. 3 and No. 4 denote the *clpB*-JD-F/R amplification products of the USA300Δ*clpB* strain and the USA300 wild-type strain, respectively. Product 1 in Figure 1B underwent sequencing, and the results are presented in Supplementary material 1. The construction process of the complemented strain of *clpB* is shown in Supplementary material 1. In the right figure of Figure 1B, the complemented strain was successfully constructed.

### 3.2 Changes in the growth curves of MRSA USA300 Δ*clpB*, CΔ*clpB*, Δ*clpB*-pCM, and wild-type strains

**Figure 2:**
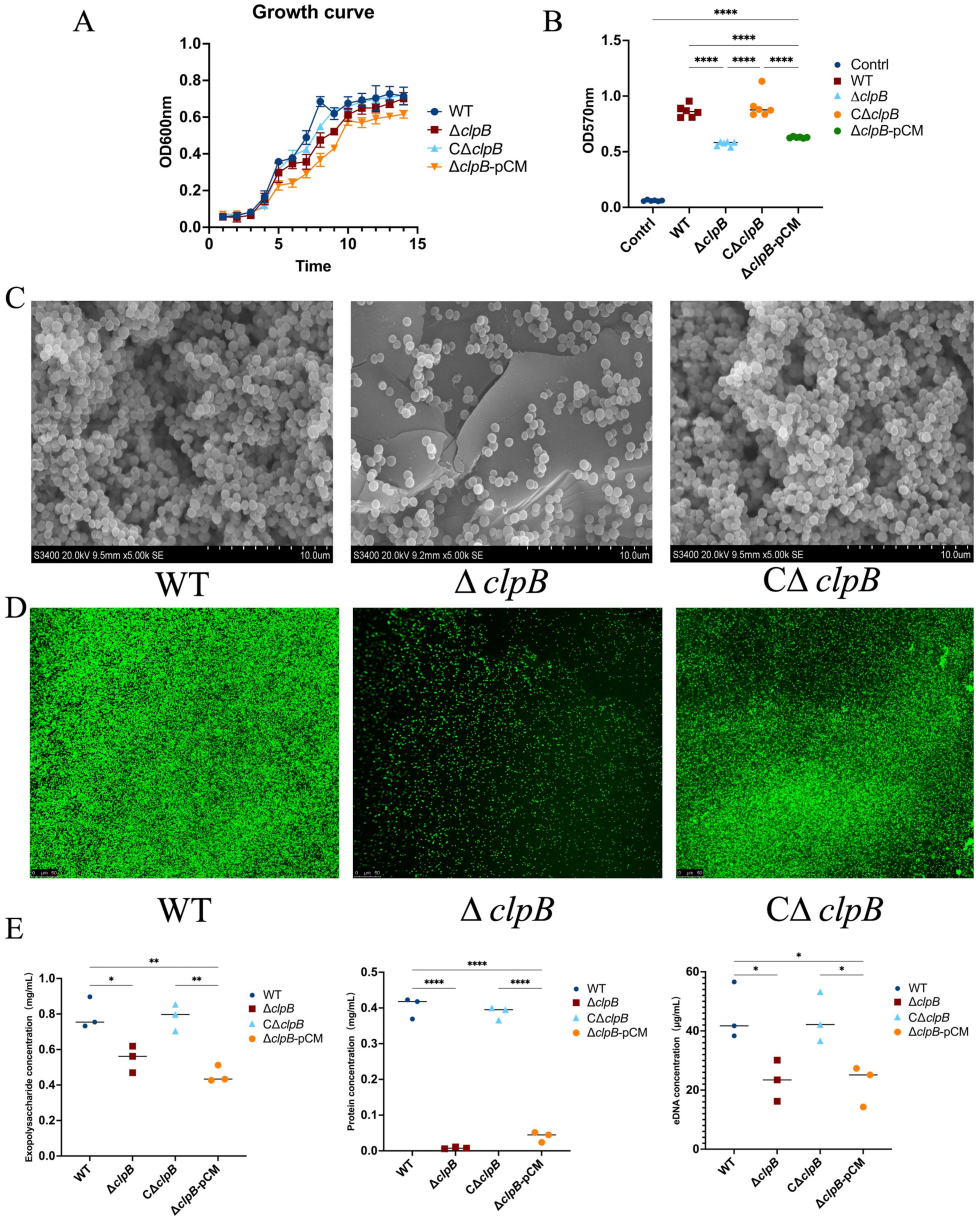
Effect of *clpB* gene on biofilm formation (* *p* < 0.05, ** *p* < 0.01, *** *p* < 0.001, **** *p* < 0.0001). **(A)** Growth curves of Δ*clpB*, CΔ*clpB,* Δ*clpB*-pCM and wild-type strains; **(B)** Crystal violet staining of Δ*clpB*, CΔ*clpB,* Δ*clpB*-pCM and wild-type strains, control: Sterile TSB solution; **(C)** Scanning electron microscopy of Δ*clpB*, CΔ*clpB* and wild-type strains; **(D)** Confocal laser scanning microscopy of Δ*clpB*, CΔ*clpB* and wild-type strains; **(E)** Changes of extracellular matrix (extracellular protein, extracellular polysaccharide, eDNA) among Δ*clpB*, CΔ*clpB,* Δ*clpB*-pCM and wild-type strains.

As shown in Figure 2A, during the 1–14 h culture period, the OD600 values of Δ*clpB*, CΔ*clpB,* Δ*clpB-*pCM and wild-type strains showed a continuous upward trend over time, indicating a simultaneous increase in cell density and cell quantity. After 14 h, there was no significant change in the OD600 values of these strains (*p* > 0.05).

### 3.3 The Δ*clpB* strain has defects in biofilm formation

Biofilm formation by MRSA USA300 and its Δ*clpB,* CΔ*clpB* and Δ*clpB-*pCM strains was assessed using crystal violet staining. Figure 2B demonstrates a significant inhibition of biofilm formation in the Δ*clpB* strains compared to the wild-type strains (*p* < 0.0001). By constructing CΔ*clpB* and Δ*clpB*-pCM strains, we found that the biofilm formation of the CΔ*clpB* strains was like that of the wild-type strains. However, compared with the Δ*clpB* strains, the biofilm formation of the CΔ*clpB* strains changed significantly and increased substantially (*p* < 0.0001). The results of Δ*clpB*-pCM were consistent with those of the Δ*clpB* strain, which supported the success of the CΔ*clpB* strain and confirmed the impact of the Δ*clpB* strain on the biofilm. Scanning electron microscopy analysis (Figure 2C) revealed distinct differences in biofilm morphology between the three strains. The wild-type strain exhibited dense and surface-adherent biofilm structures, distinct from planktonic bacteria, forming extensive bacterial aggregates indicative of mature biofilms. Conversely, the Δ*clpB* strain displayed sparse bacterial adhesion to the surface, disrupted three-dimensional biofilm structures, and an inability to form mature biofilms. After constructing the CΔ*clpB* strain, we found that the biofilm morphology of the CΔ*clpB* strain had returned to a density like that of the wild-type strain, and its biofilm morphological structure presented a three-dimensional form, which was significantly different from that of the Δ*clpB* strain. Furthermore, confocal laser microscopy (Figure 2D) was employed to assess biofilm integrity. The wild-type strain emitted intense green fluorescence (left image), indicating intact cell membranes and concentrated green fluorescence. In contrast, the Δ*clpB* strain exhibited minimal green fluorescence, suggesting compromised biofilm integrity due to *clpB* gene deletion. Subsequently, *clpB* was replenished and it was found to exhibit a strong green fluorescence, indicating that gene complement restored its missing function. In conclusion, the deletion of the *clpB* gene significantly impacts biofilm formation.

### 3.4 Changes of biofilm matrix of MRSA USA300 Δ*clpB*, CΔ*clpB*, and wild-type strains

The analysis of biofilm components in the Δ*clpB*, CΔ*clpB*, Δ*clpB-*pCM and wild-type strains revealed notable differences, as illustrated in Figure 2E. Specifically, a decrease in extracellular polysaccharide, eDNA, and extracellular protein contents within the biofilm matrix was observed in the Δ*clpB* strain compared to the wild-type strain (*p* < 0.05, *p* < 0.05, and *p* < 0.0001, respectively). Notably, the protein content exhibited a significant reduction (*p* < 0.0001). To confirm the changes in the extracellular matrix of bacteria caused by the Δ*clpB* strain, we replenished the genes of the mutant strain and found that the trend of change was roughly like that before the mutation. The above results explain the regulatory effect of *clpB* on biofilms.

### 3.5 Effect of MRSA USA300 Δ*clpB* and wild-type strains on skin infection in mice

**Figure 3:**
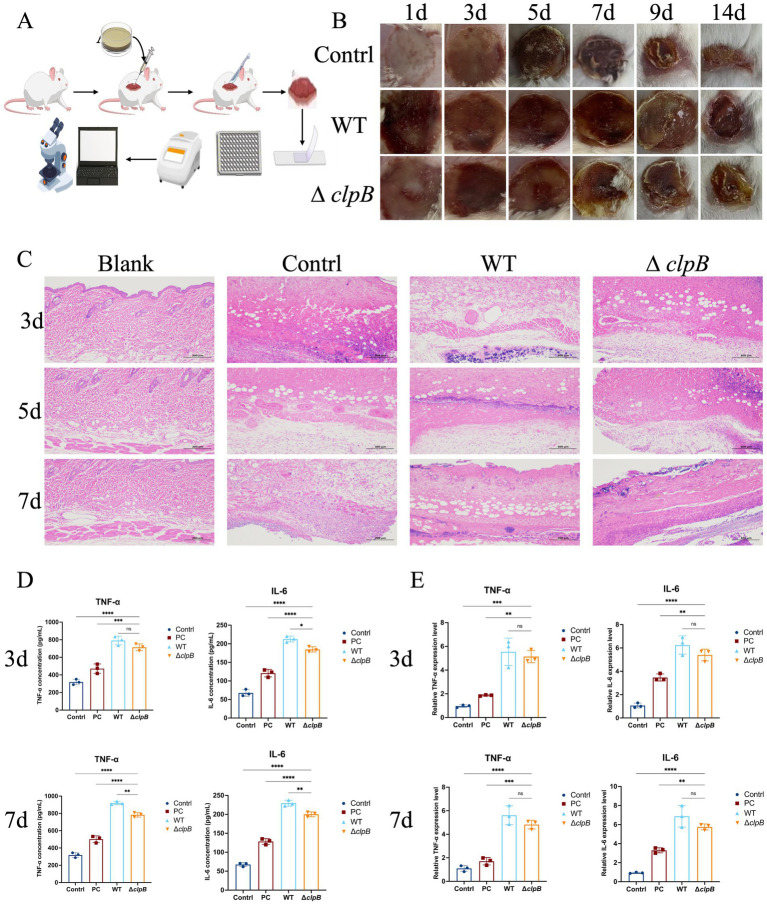
Effect of *clpB* gene on skin infection in mice (* *p* < 0.05, ** *p* < 0.01, *** *p* < 0.001, **** *p* < 0.0001). **(A)** Mouse experiment diagram; **(B)** Wound surface of control group, wild-type group and Δ*clpB* group at 1, 3, 5, 7, 9, and 14 days. Control group: only burns. **(C)** Pathological sections of blank group, control group, wild-type and Δ*clpB* group at 3, 5, and 7 days. Blank group: normal skin; control group: only burns. **(D)** The concentration changes of TNF-*α* and IL-6 in control group, PC group, wild-type group and Δ*clpB* group at 3 and 7 days. **(E)** The expression levels of TNF-α and IL-6 in control group, PC group, wild-type group and Δ*clpB* group at 3 and 7 days. Control group: only burns; PC group: Vancomycin treatment group.

Animal experiments were conducted following the experimental design outlined in Figure 3A. post-burn injuries in mice resulted in circular wounds on their dorsal area, accompanied by slight exudation and edema in the surrounding tissues. The wounds exhibited gray and white discoloration with visible coagulated necrotic tissue, characterized by a firm texture and limited mobility. The burned skin showed a notable reduction in local extension, increased resistance to pulling, and a distinct white border surrounding the burn wound. Additionally, the skin at the wound periphery appeared slightly elevated compared to the adjacent normal skin, with a clearly demarcated boundary. In contrast, the control group depicted in Figure 3C exhibited intact epidermal layers with well-defined structural organization. The dermal collagen fibers displayed a regular and intertwined pattern, along with the presence of skin appendages such as hair follicles and sebaceous glands distributed throughout. Subcutaneous tissue beneath the dermis consisted of loose connective tissue and muscle layers, devoid of significant inflammatory cell infiltration. Histological examination of skin samples from the control group with second-degree burns (Figure 3C) revealed extensive necrosis across all layers, characterized by abundant pale pink homogeneous material and necrotic cell fragments. Skin appendages were sparse, and localized edema with scattered inflammatory cells was observed in the subcutaneous region.

Following the establishment of the burn and scald model, subcutaneous injection of wild and Δ*clpB* strains was performed to induce the skin infection model, as depicted in Figure 3B. The experimental groups consisted of the burn and scald control group, the wild-type strain group (WT), and the Δ*clpB* strain group (∆*clpB*). On the initial day of the experiment, mice exhibited suppurative secretions, peripheral tissue edema, wound redness, and inflammation at the back wounds, with some animals showing blood clots. Compared to the control group, both the wild-type strain and Δ*clpB* strain groups displayed more severe wound injuries. By the third day, mice exhibited overall good health, with wounds forming pale yellow scabs characterized by complete skin coverage, a rough and firm texture, tight adherence to the wound bed, and disappearance of the white halo around the wound edges. In both the wild-type and Δ*clpB* strain groups, purulent secretions, increased redness and swelling, and more pronounced local blood clots were observed compared to the first day. By the fifth day, mice resumed normal activities. While the control group showed raised scab edges detached from the skin surface, the wild-type strain and Δ*clpB* strain groups still exhibited purulent secretions on the wound surface with minimal changes. By the seventh day, mice were in good health, with all wounds and scabs showing noticeable warping of the scab edges. The control group exhibited reduced wound areas compared to the fifth day. On the ninth day, mice remained in good health, with significant reduction in wound areas, initiation of scab detachment, thickening and hardening of residual scabs to a light yellow-brown color, and exposure of new red skin tissue. Hair regeneration was observed around the wound margins, further reducing the wound area compared to the seventh day. By the 14th day, mice displayed normal eating and activity patterns. Wounds in all three groups had significantly decreased, with the control group showing the most advanced healing. Although the wild-type strain and Δ*clpB* strain groups exhibited smaller wounds than before, scab shedding was incomplete. In conclusion, continuous monitoring revealed that the wound recovery in the Δ*clpB* and wild-type strain groups was inferior to that of the control group, with the Δ*clpB* strain group showing better recovery than the wild-type strain group. This indicates that gene deletion influences the severity of skin infection and the wound healing process.

Visible changes in the wound surface were assessed by examining skin tissue alterations, conducting colony counting on a portion of the tissue, and selecting a dilution of 10^−6^ (plate colony counting within the range of 30–300) after a series of dilutions. The bacterial concentration in the wild-type strain group was 1.35*10^9^ CFU/mL (*n* = 3), while in the Δ*clpB* strain group, it was 7.5*10^8^ CFU/mL (*n* = 3) (refer to Supplementary material S2). Pathological biopsies were taken from some tissues for HE detection (see Figure 3C). On the third day, skin tissues from the control group exhibited extensive full-layer necrosis, pale pink homogenates, deposition of necrotic cell fragments, and a significant reduction in skin appendages such as hair follicles and sebaceous glands. Subcutaneous tissue displayed slight edema with sporadic lymphocyte infiltration. Skin tissue from the wild-type strain group also showed pale pink homogenates and necrotic cell debris, along with mild edema and lymphocyte infiltration in the subcutaneous tissue. In contrast, the Δ*clpB* strain group exhibited less skin tissue edema and fewer inflammatory cells and lymphocytes compared to the wild-type group. By the fifth day, the control group still displayed pale pink homogenates and necrotic cell fragments in the skin tissue, with no significant changes in edema and lymphocyte infiltration in the subcutaneous tissue compared to the third day. The wild-type strain group showed more apparent subcutaneous tissue edema and lymphocyte infiltration. The Δ*clpB* strain group exhibited smaller areas of subcutaneous tissue edema and lymphocyte infiltration compared to the wild-type group. On the seventh day, the control group continued to exhibit extensive full-layer necrosis in the skin tissue, along with pale pink homogenates and necrotic cell fragments. The subcutaneous tissue displayed focal connective tissue hyperplasia, scattered inflammatory cell infiltration, and increased neovascularization. The skin tissue of the wild-type strain group also showed extensive full-layer necrosis, pale pink homogenates, necrotic cell fragments, and focal necrotic shedding. The Δ*clpB* strain group had significantly fewer lymphocyte infiltrates in the skin tissue compared to the wild-type group. Overall, HE staining indicated a more severe infection in the wild-type group than in the Δ*clpB* strain group.

Subsequently, we assessed the expression and transcriptional levels of the inflammatory cytokines IL-6 and TNF-*α* in the control group, the vancomycin-treated positive control group, the wild-type strain infection group, and the Δ*clpB* strain infection group to evaluate the inflammatory response during wound healing. IL-6 and TNF-*α* are known to increase vascular permeability and promote tissue edema, thereby impairing the wound repair process. As sensitive markers of systemic inflammation, their elevated concentrations correlate positively with the severity of the inflammatory response. ELISA results (Figure 3D) demonstrated that on day 3 of the experiment, the concentrations of TNF-*α* and IL-6 in the Δ*clpB*, WT, and PC groups were significantly elevated compared to the control group (*p* < 0.0001). When compared with the PC group, both the WT and Δ*clpB* groups exhibited significantly higher levels of TNF-*α* and IL-6 (TNF-*α*: *p* < 0.001; IL-6: *p* < 0.0001). In comparison to the WT group, the Δ*clpB* group showed a reduction in TNF-α levels, although this difference did not reach statistical significance (*p* > 0.05), whereas IL-6 levels were significantly decreased (*p* < 0.05). By day 7, TNF-*α* and IL-6 levels in the Δ*clpB*, WT, and PC groups remained significantly higher than those in the control group (*p* < 0.0001). Furthermore, relative to the PC group, TNF-*α* and IL-6 levels were significantly increased in both the WT and Δ*clpB* groups (*p* < 0.0001). However, the Δ*clpB* group displayed significantly lower levels of both cytokines compared to the WT group (*p* < 0.01).

Real-time fluorescent quantitative PCR was employed to assess changes in TNF-*α* and IL-6 mRNA levels in infected skin under various conditions (Figure 3E). On day 3, compared with the control group, the expression levels of TNF-α and IL-6 were significantly elevated in the Δ*clpB*, WT, and PC groups (TNF-α: *p* < 0.001; IL-6: *p* < 0.0001). Moreover, the expression levels of both cytokines in the Δ*clpB* and WT groups were significantly higher than those in the PC group (*p* < 0.01). Although the expression levels of TNF-α and IL-6 in the Δ*clpB* group were lower than those in the WT group, the differences did not reach statistical significance (*p* > 0.05). On day 7, the expression levels of TNF-α and IL-6 remained significantly higher in the Δ*clpB*, WT, and PC groups compared to the control group (*p* < 0.0001). Additionally, both the Δ*clpB* and WT groups exhibited significantly higher cytokine expression than the PC group (*p* < 0.001 for TNF-α; *p* < 0.01 for IL-6). Consistent with day 3, the Δ*clpB* group showed lower TNF-α and IL-6 expression than the WT group, though these differences were not statistically significant (*p* > 0.05).

The findings regarding TNF-α and IL-6 levels and expression indicate that *clpB* mutations potentially modulate inflammation by augmenting the inflammatory response compared to the control group, albeit to a lesser extent than the wild-type strain group, suggesting a time-dependent regulatory impact.

## 4 Discussion

Bacterial ClpB, an ATP-dependent depolymerase categorized within the Hsp100/*Clp* subfamily of AAA + ATPases, collaborates with DnaK to revive polymeric proteins, enhancing bacterial survival in harsh environmental conditions like heat and oxidative stress. It is prevalent in bacteria, protozoa, fungi, and plants but notably absent in animals and humans (Kędzierska-Mieszkowska and Zolkiewski, 2021; Kędzierska-Mieszkowska and Arent, 2020). This absence in human cells underscores the potential of ClpB as a promising target for innovative antimicrobial approaches, crucial in combating the escalating issue of antibiotic resistance among pathogenic bacteria, thereby offering novel avenues for investigating microbial infections. It is known that ClpB, as a molecular chaperone, plays a key role in protein quality control and depolymerization of aggregated proteins (Kędzierska-Mieszkowska and Zolkiewski, 2021). The formation of biofilms involves the synthesis and secretion of many proteins. ClpB may indirectly affect the development and maturation of biofilms by ensuring the correct folding and function of some key proteins. Meanwhile, previous research has elucidated the significance of ClpB in various microbes such as *Escherichia coli*, *Helicobacter pylori*, and *Leptospira* (Kędzierska-Mieszkowska and Zolkiewski, 2021; Kędzierska-Mieszkowska and Arent, 2020; Alam et al., 2021). While most studies have concentrated on gram-negative bacteria, limited attention has been given to the biofilm formation of Gram-positive bacteria like MRSA. This study delves into the biofilm formation of MRSA, shedding light on the regulatory function of ClpB within biofilms.

In our prior investigation, we identified that the modulation of biofilm formation in response to drug treatment is primarily associated with the downregulation of the ClpB protein (Miao et al., 2024). To further explore the research potential of ClpB, we employed homologous recombination gene knockout techniques to disrupt its gene and generate a Δ*clpB* strain. Comparative analysis between the Δ*clpB* and wild-type strains revealed distinct differences in bacterial growth kinetics, with the Δ*clpB* strains exhibiting slower growth rates. This decelerated growth may impact the virulence of MRSA, consequently impeding growth and significantly suppressing biofilm formation in the MRSA USA300 strain. Immediately after, we complemented *clpB* to obtain the CΔ*clpB* strain. As shown in Figure 2A, the growth trend of the CΔ*clpB* strain was consistent with that of the wild-type strain. In comparison to the Δ*clpB* strain, the CΔ*clpB* strain exhibited a distinct growth profile during the early to mid-phase, though the curves eventually converged in the later stage. We speculate that while the deletion of *clpB* may impair certain bacterial functions and lead to slowed growth, it does not completely inhibit proliferation. Therefore, to determine whether *clpB* influences biofilm formation independently of growth, we proceeded with further experiments. Utilizing crystal violet staining, scanning electron microscopy (SEM), and confocal laser microscopy, we observed a marked reduction in the biofilm-forming capacity of the Δ*clpB* strain, underscoring the pivotal role of the *clpB* gene in the biofilm formation process (Figures 2B–D). Notably, the biofilm architecture of the Δ*clpB* strain displayed significant alterations compared to that of the wild-type strain. While the wild-type strain exhibited a compact structure with strong adhesion capable of forming mature biofilms, the Δ*clpB* strain displayed a loose structure with weak adhesion, precluding the formation of mature biofilms. Confocal laser microscopy further revealed compromised biofilm integrity in the Δ*clpB* strain, as evidenced by a substantial reduction in green fluorescence, indicating that *clpB* gene deletion adversely impacted biofilm stability. Three experiments related to biofilms were conducted, and the results demonstrated the differences between wild-type strains and Δ*clpB* strains. To verify the results of Δ*clpB* strains, experiments on CΔ*clpB* strains were carried out, as well as crystal violet staining, scanning electron microscopy, and confocal laser microscopy. The results further confirmed the impact of Δ*clpB* strains on biofilms. Previous research has highlighted the crucial role of *Clp* proteins not only in biofilm formation and bacterial-mucus interactions but also in general stress regulation (Mao et al., 2024). Additionally, studies have implicated proteins such as GroEL, DnaK, and ClpB in mediating responses to oxidative stress, antioxidant defense mechanisms, biofilm formation, production of toxic enzymes, bacterial adhesion, capsule formation, and antibiotic resistance (Abirami et al., 2023). Therefore, in conjunction with our experimental findings, it is evident that ClpB is intricately involved in biofilm formation, significantly influencing the robustness of MRSA biofilms and its pathogenicity. Zhao et al. (2015) have similarly demonstrated that the absence of *clpB* disrupts biofilm formation, corroborating our results. In contrast, our research still differs in terms of target organisms, clinical relevance, and the functional mechanism of ClpB. Our current study emphasizes that as a bacteria-specific molecular chaperone (not present in humans), ClpB is a potential target for novel anti-biofilm therapies targeting MRSA. The potential mechanism by which ClpB, acting as a chaperone protein, contributes to biofilm formation may involve the regulation of bacterial stress responses or indirect modulation of bacterial virulence factors and extracellular matrix components (e.g., polysaccharides, proteins, DNA).

Extracellular Polymeric Substances (EPS) constitute the primary component of bacterial biofilms, with key constituents including DNA, proteins, and extracellular polysaccharides crucial for biofilm structural integrity and cellular protection (Wang et al., 2022). To validate this proposition, an analysis of extracellular matrix components was conducted. Experimental findings revealed that the deletion of the *clpB* gene altered the composition of biofilm matrix components, notably leading to a significant reduction in protein content. Subsequently, we conducted extracellular matrix component analysis experiments on the CΔ*clpB* strain and found that the results further confirmed the changes in the extracellular matrix of the biofilm caused by the Δ*clpB* strain. This observation suggests that the absence of the ClpB protein may induce misfolding in other bacterial proteins, thereby impacting their synthesis, secretion, and functionality. Research indicates (Campoccia et al., 2021) that extracellular DNA (eDNA) plays a role in bacterial aggregation, facilitating intercellular adhesion, surface attachment, bacterial anchoring, and biofilm structure stabilization through interweaving various polymer components. Extracellular proteins are pivotal for biofilm formation and pathogenicity, contributing to adhesion resistance, structural maintenance, and immune evasion, rendering biofilms resilient to eradication. Extracellular polysaccharides, particularly PIA/PNAG, are essential for biofilm development, mediating initial adhesion, structural reinforcement, immune evasion, and antimicrobial resistance. Experimental data demonstrated that *clpB* gene deletion resulted in decreased levels of exopolysaccharides, eDNA, and extracellular proteins. It is hypothesized that ClpB may indirectly influence the expression of *icaADBC* by modulating *icaR* (a PIA synthesis inhibitor) or *SigB* (a stress response *σ* factor), thereby impacting polysaccharide production. ClpB aids in correcting protein misfolding and potentially maintains biofilm integrity by stabilizing the folding of the key extracellular matrix (ECM) protein Bap, hence, ClpB mutations could affect biofilm formation. ClpB may enhance biofilm structure by modulating autolysin activity (e.g., AtlA), influencing bacterial autolysis and eDNA release. This speculation provides a basis for further mechanistic investigations.

Upon entering a host, bacteria encounter diverse environmental challenges such as fluctuations in temperature, osmotic pressure, and pH. To cope with these stimuli, bacteria modulate the expression of virulence factors and stress response proteins, including heat shock proteins and chaperones (Wang et al., 2022). Among these proteins, ClpB is a crucial component of the microbial stress response machinery, acting to prevent protein aggregation and facilitate the refolding of denatured proteins. In this investigation, we assessed the impact of ClpB on inflammatory responses (TNF-*α*, IL-6) and wound healing by comparing the Δ*clpB* group with the wild-type strain at various time points. Our findings, as depicted in Figures 3A–E and supported by histological (HE staining) and molecular (ELISA and RT-qPCR) analyses, revealed that the absence of *clpB* led to a dampened inflammatory reaction in mice, characterized by reduced inflammatory cell infiltration and diminished expression of IL-6 and TNF-α. These results suggest that ClpB may modulate biofilm formation and host immune responses through the regulation of bacterial stress responses and virulence determinants. Our observations align with previous research indicating (Alam et al., 2021) the pivotal role of ClpB from various bacterial species in survival and pathogenicity in experimental models subjected to diverse stress conditions.

This study reveals the significance of ClpB as a key regulator in MRSA biofilm formation, offering preliminary insights for future investigations into effective anti-biofilm agents. Through homologous recombinant gene knockout, the *clpB* gene was targeted, resulting in a strain with the gene deletion. Comparative analysis of biochemical and molecular profiles pre- and post-ClpB knockout unveils a novel mechanism for elucidating the interplay between specific genes and biofilm development. Subsequent *in vitro* and *in vivo* experiments were conducted to validate this relationship. Nonetheless, limitations exist in the current study, including potential impacts of ClpB knockout on other bacterial physiological functions, necessitating further investigation into its specificity. Additionally, our study demonstrates that the deletion of the *clpB* gene does influence biofilm formation, although the effect is not as pronounced as that observed with the deletion of the *sarA* gene (Kim et al., 2022; Valliammai et al., 2020). Therefore, we speculate that in the biofilm regulatory network of MRSA, SarA may be a core global regulatory factor located further upstream, while ClpB may act as a cofactor, exerting its influence on the stability or function of certain key proteins in the SarA regulatory pathway. Another possibility is that ClpB and SarA jointly regulate biofilms through parallel but complementary pathways. However, when considering our experimental results in conjunction with those involving the complementation strain, a regulatory role of ClpB in biofilm development becomes evident. Notably, ClpB is absent in humans and mammals, making it a promising and specific target for anti-biofilm therapeutic strategies. Future research endeavors will delve into the precise mechanisms of ClpB, including assessing the repercussions of ClpB knockout on bacterial gene expression and protein functionality via proteomic or transcriptomic analyses. Additionally, exploration of ClpB’s role across diverse bacterial species or infection models is warranted. However, unlike previous studies mainly based on correlation analysis, this study provided direct genetic evidence in MRSA by constructing Δ*clpB* and CΔ*clpB* equivalent gene strains, demonstrating that ClpB may be a factor in biofilm formation and pathogenicity *in vivo*. This provides a theoretical basis for developing anti-MRSA biofilm strategies targeting ClpB.

## 5 Limitations and prospects

This study reveals the key role of ClpB in the formation of MRSA biofilms, but there are still some limitations. Firstly, our research focuses on MRSA. The functional universality of ClpB in different bacterial species remains to be further explored. Secondly, although we have confirmed the phenotypic impact of ClpB, the specific molecular mechanisms by which it affects biofilm formation, such as how it regulates key virulence factors, are not yet fully understood. In addition, compared with the core regulatory factor *SarA*, the role of ClpB is relatively weak, indicating that it may be not a major regulator, but its in-depth role requires further investigation.

Based on these findings, future research will focus on the following directions: (1) Utilize transcriptomics and proteomics techniques to systematically clarify the biofilm-related pathway networks regulated by ClpB and (2) Verify the conservation of ClpB function in different pathogens to evaluate its potential as a broad-spectrum anti-biofilm target. Ultimately, through high-throughput screening, small molecule inhibitors targeting ClpB are sought out, and their anti-biofilm efficacy is evaluated in in vivo and *in vitro* models, laying the foundation for the development of novel anti-infection strategies.

## 6 Conclusion

In essence, the ClpB protein serves a crucial regulatory function in the biofilm formation of methicillin-resistant *S. aureus*, suggesting its viability as a prospective target for biofilm inhibition.

## Data Availability

The datasets presented in this study can be found in online repositories. The names of the repository/repositories and accession number(s) can be found in the article/Supplementary material.
