# Supplementary material for: ClpB affects biofilm formation in methicillin-resistant Staphylococcus aureus
Source: Front Microbiol. 2025 Dec 4;16:1723924. doi: 10.3389/fmicb.2025.1723924 (PMC12711867; doi:10.3389/fmicb.2025.1723924)
Supplement: Supplementary file 1 [file Data_Sheet_1.pdf]

Construction of MRSA USA 300 Δ*clpB* mutant

The upstream and downstream homologous arms were amplified by PCR

Using the extracted USA300 DNA as the template, the following reaction system was prepared with primers *clpB*-19-F and *clpB*-up-R, *clpB*-down-F and *clpB*-19-R, respectively:

Amplification of upstream and downstream homologous arms

System grouping	Reaction volume
2×pfu PCR mix	25μl
Primer P1（10μM）	2μl
Primer P2（10μM）	2μl
USA300 DNA	2μl
ddH_2O	19μl
Total	50μl

Amplification condition：94℃ 5min 30cycle（94℃ 30sec, 55℃ 30sec, 72℃ 40sec）10℃ hold on. The expanded upstream and downstream homologous arms were recovered.

Seamless clone connection

The amplified *clpB* up and *clpB* down fragments were recovered and seamlessly cloned to connect the upstream and downstream homologs Arm, the reaction system is as follows:

Amplification of upstream and downstream homologous arms

System grouping	Reaction volume
5×infusion mix	2μl
clpB-up product	3μl
clpB-down product	3μl
pUC19[EcoRI-hindIII] Linearized carrier	2μl

Total	10μl

Placed on ice for 30min, the DH5α receptive state was chemically transformed, the Amp resistant plate was coated, and the white spot clones were cultured at 37℃ for sequencing.

Knockout vector construction

The carrier framework was prepared with primers *clpB*-pKOR1-F and *clpB*-pKOR1-R as the template plasmid clpB-ud-puc19, and the reaction system was prepared as follows：

The carrier frame reaction system was prepared

System grouping	Reaction volume
2×Superpfu PCR mix	25μl
clpB-pKOR1-F（10μM）	2μl
clpB-pKOR1-R（10μM）	2μl
clpB-ud-puc19	1μl
ddH_2O	20
Total	50μl

Amplification program 94℃ 5min   32Cycle（94℃ 30sec, 55℃ 30sec, 68℃ 1min） 10℃ hold on. The amplified products were purified by PCR products.

Using primer PKOR1-*clpB*-F/PKOR1-*clpB*-R to backexpand pKOR1 as a carrier, the reaction system was prepared as follows:

The carrier frame reaction system was prepared

System grouping	Reaction volume
2×Superpfu PCR mix	25μl
pKOR1-*clpB*-F（10μM）	2μl
pKOR1-*clpB*-R（10μM）	2μl
pKOR1	1μl
ddH_2O	20
Total	50μl

Amplification program 94℃ 5min 32Cycle（94℃ 30sec, 55℃ 30sec, 68℃ 3min）10℃ hold on. The amplified products were purified by PCR products.

Seamless clone connection

By seamlessly cloning the upstream and downstream homologous arm fusion fragment and the prepared pKOR1 backexpansion product, the reaction system is as follows:

Seamless clone connection

System grouping	Reaction volume
5×infusion mix	1μl
clpB ud Amplified product	3μl
pKOR1 Back expansion product	1μl
Total	5μl

The DH5α receptive state was chemically transformed after 30min on ice, and the Amp resistant plate was coated for screening.

Knockout vector electrical conversion RN4220 and RN4220 phage transduction

pKOR1-*clpB* ud plasmid containing upstream and downstream homologous arm sequences of *clpB* was added to the prepared RN4220 receptor cells for electrotransformation at 2300KV and was coated with TSA (Cm 5μg/ml) plate for culture at 30℃. RN4220-*clpB*-pKOR1 phage was prepared, USA300 was transformed by phage transduction method, and cultured at 30℃ on TSA plate coated with Cm 10μg/ml.

Mutant screening and identification

The positive clones were inoculated with 5ml TSB (Cm 10μg/ml) liquid medium at 30℃, transferred to fresh 5ml TSB (Cm 10μg/ml) overnight at 43℃ the next day, and then coated with TSA (Cm7.5μg/ml) plate at 43℃. Selected clones were transferred to fresh 5ml TSB (Cm 5μg/ml) overnight culture at 43℃, coated with TSA plate at 30℃, selected clones were transferred to 5ml TSB culture at 30℃, the bacterial solution was diluted and coated on ATC (1μg/ml) plate, and the clones grew. Dots were placed on TSA plate and TSA (Cm 10μg/ml) plate, and short clones of chloramphenicol plate

were selected for identification. Identification primers: *clpB*-JD-F/ *clpB*-JD-R, *clpB*-ter-F/*clpB*-ter-R.

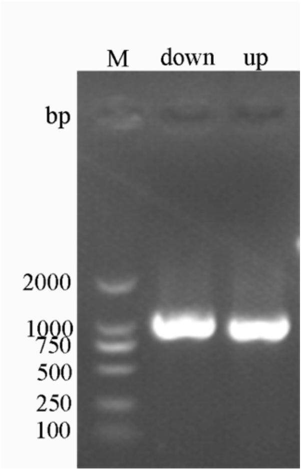

Figure S1: The upstream and downstream homologous arm of clpB was amplified by 1% agarose gel electrophoresis

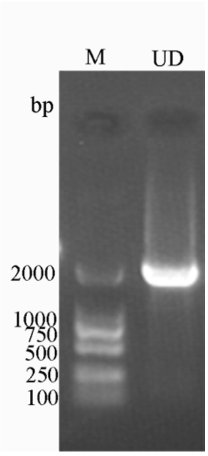

Figure S2: Agarose gel electrophoresis by amplification of clpB-pKOR1

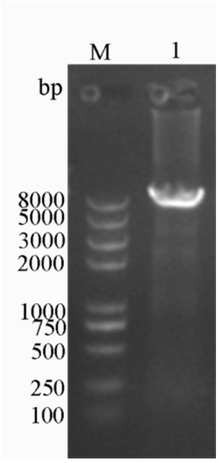

Figure S3: pKOR1 amplified agarose gel electrophoresis assay

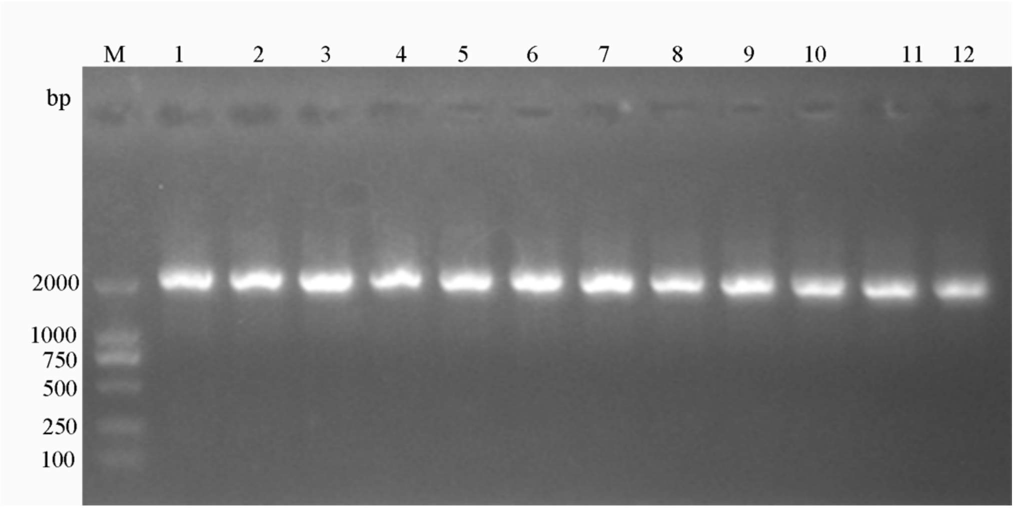

Figure S4: PCR identification of colony constructed by clpB knockout vector

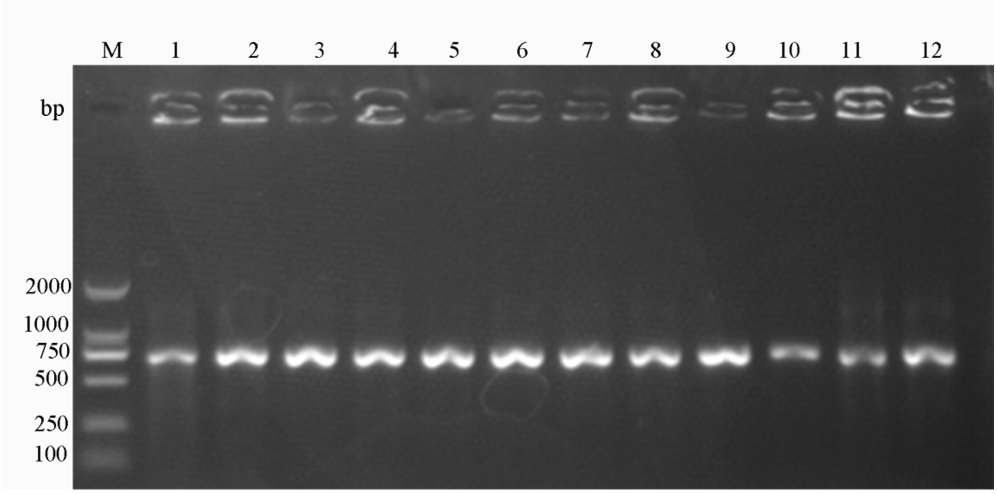

Figure S5: PCR identification of RN4220 colony by knockout vector transformation

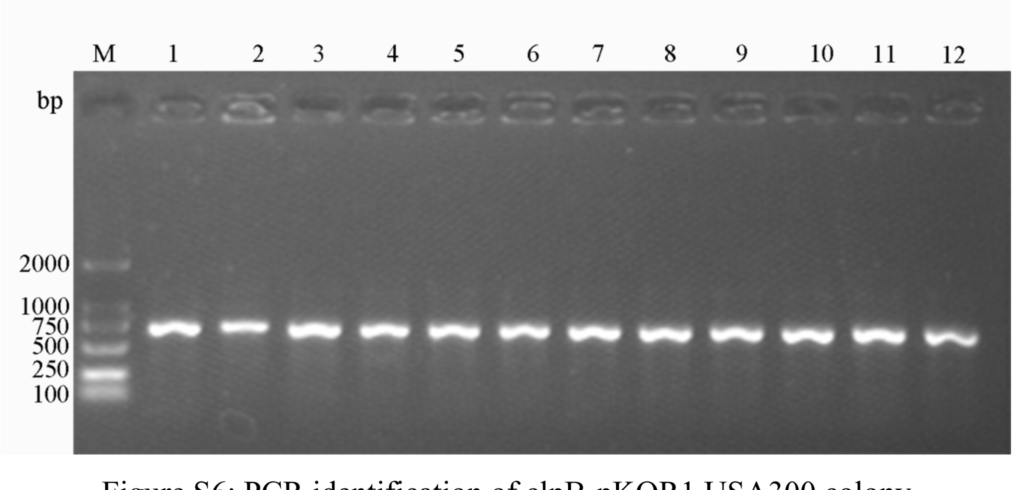

Figure S6: PCR identification of clpB-pKOR1 USA300 colony

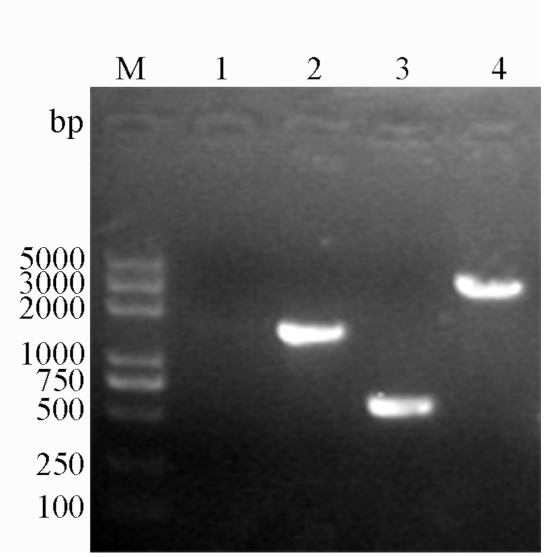

Figure S7: clpB-JD-F/R, clpB-ter-F/R wild type identification and knock-out bacteria amplification detection

clpb knockout strain sequencing

Splicing results:

TGACGGTGTTCATTTAGAGTACAAAGGAGTCCTAGCTTTAAAAGATGAAATATTAAAAGCACTTAAAAA
GAAATAACATATGTTTAATAAGTCTAGTTAATGTGTAACGTAACATTAGCTAGATTTTTTTATTCAAAAAA
ATATTTACAAATATTAGGAAATTTAAGTGTAAAAGAGTTGATAAATGATTATATTGGGACTATAATATAATT
AAGGTCAAAGAAAGTCAAAGTCAAAAGTGCAACTTGACCAACTCTACAAATTAGAGGTGAAATTATAA
TTGAACCAAGAATGTGATGATTAATCGCATTCTTGGTTTTTTAGATTTGAAATTTATTATAATTGTTCATTG
GCAATATACTTTTTAAATGATTTAATAAACACCAATATTTCTGGGCTTTCTTTTTTGCTGTATATATATGTAA
ATGAAATAGGTGCTTGTAAAATTTTCGTGTTAATAACCGAAATATTGTAATCACTATTTGTTGTTATATAAA
GAGGCAGGAATGATATACCTTGATTCATTTCGATTAATTTAATTGAAGTATGCACATCATTGATAGATAGA
AATTGTGCTTTTTCATAAATATTTAAAATATTATTTTTAAGTGATGACCAATATTCTGGATGGTTATCAACT
TATTAA

clpb knockout comparison:

```
Fast alignment of DNA sequences DNAMAN2 and DNAMAN1

Ktuple=2  Gap_penalty=7

Upper line: DNAMAN2, from 984 to 1627
Lower line: DNAMAN1, from 2 to 646

Upper line: DNAMAN2, from anticipate
Lower line: DNAMAN1, from sequencing


DNAMAN2:DNAMAN1 identity= 99.53%(642/645) gap=0.15%(1/646)
983  AGACGGTGTACATTTAGAGTACAAAGGAGTCCTAGCTTTAAAAGATGAAATATTAAAAGC
      |||||||| ||||||||||||||||||||||||||||||||||||||||||||||||||
1    TGACGGTGTTCATTTAGAGTACAAAGGAGTCCTAGCTTTAAAAGATGAAATATTAAAAGC

1043 ACTTAAAAAGAAATAACATATGTTTAATAAGTCTAGTTAATGTGTAACGTAACATTAGCT
     ||||||||||||||||||||||||||||||||||||||||||||||||||||||||||||
61   ACTTAAAAAGAAATAACATATGTTTAATAAGTCTAGTTAATGTGTAACGTAACATTAGCT

1103 AGATTTTTTTATTCAAAAAAATATTTACAAATATTAGGAAATTTAAGTGTAAAAGAGTTG
     ||||||||||||||||||||||||||||||||||||||||||||||||||||||||||||
121  AGATTTTTTTATTCAAAAAAATATTTACAAATATTAGGAAATTTAAGTGTAAAAGAGTTG
```

```
1163 ATAAATGATTATATTGGGACTATAATATAATTAAGGTCAAAGAAAGTCAAAGTCAAAAGT
     ||||||||||||||||||||||||||||||||||||||||||||||||||||||||||||
181  ATAAATGATTATATTGGGACTATAATATAATTAAGGTCAAAGAAAGTCAAAGTCAAAAGT

1223 GCAACTTGACCAACTCTACAAATTAGAGGTGAAATTATAATTGAACCAAGAATGTGATGA
     ||||||||||||||||||||||||||||||||||||||||||||||||||||||||||||
241  GCAACTTGACCAACTCTACAAATTAGAGGTGAAATTATAATTGAACCAAGAATGTGATGA

1283 TTAATCGCATTCTTGGTTTTTTAGATTTGAAATTTATTATAATTGTTCATTGGCAATATA
     ||||||||||||||||||||||||||||||||||||||||||||||||||||||||||||
301  TTAATCGCATTCTTGGTTTTTTAGATTTGAAATTTATTATAATTGTTCATTGGCAATATA

1343 CTTTTTAAATGATTTAATAAACACCAATATTTCTGGGCTTTCTTTTTTGCTGTATATATA
     ||||||||||||||||||||||||||||||||||||||||||||||||||||||||||||
361  CTTTTTAAATGATTTAATAAACACCAATATTTCTGGGCTTTCTTTTTTGCTGTATATATA

1403 TGTAAATGAAATAGGTGCTTGTAAAATTTTCGTGTTAATAACCGAAATATTGTAATCACT
     ||||||||||||||||||||||||||||||||||||||||||||||||||||||||||||
421  TGTAAATGAAATAGGTGCTTGTAAAATTTTCGTGTTAATAACCGAAATATTGTAATCACT

1463 ATTTGTTGTTATATAAAGAGGCAGGAATGATATACCTTGATTCATTTCGATTAATTTAAT
     ||||||||||||||||||||||||||||||||||||||||||||||||||||||||||||
481  ATTTGTTGTTATATAAAGAGGCAGGAATGATATACCTTGATTCATTTCGATTAATTTAAT

1523 TGAAGTATGCACATCATTGATAGATAGAAATTGTGCTTTTTCATAAATATTTAAAATATT
     ||||||||||||||||||||||||||||||||||||||||||||||||||||||||||||
541  TGAAGTATGCACATCATTGATAGATAGAAATTGTGCTTTTTCATAAATATTTAAAATATT

1583 ATTTTTAAGTGATGACCAATATTCTGGATGGTTATC.ACTTATTATTTGTATTTTTCAA
     |||||||||||||||||||||||||||||||||||| ||||||||
601  ATTTTTAAGTGATGACCAATATTCTGGATGGTTATCAACTTATTAA
```

Construction of MRSA USA 300 Δ*clpB* complement strain

Primer design

Primer name	Primer sequence
pCM-his-F	CACCACCACCACCACCACTAAGAATTCGTAATCATGTCAT
pCM-*clpB*-R	AGACTTATTAAACATATGGACACAGTGATTGTATTTCTGG
clpB-pCM-F	AAATACAATCACTGTGTCCATATGTTTAATAAGTCTAGTT
clpB-his-R	GTGGTGGTGGTGGTGGTGTTCATGAATTTTTTCAACATTAA
clpB-CX-F	TTCGGCATTAGAGCGTCGTTTCC
M13-R	AGCGGATAACAATTTCACACAGG

Construction of the complement carrier

Using USA300 DNA as the template, the *clpB* fragment was amplified with the primers *clpB*-pCM-F/*clpB*-his-R

System grouping	Reaction volume
2×pfu PCR mix	25μl
Primer P1（10μM）	2μl
Primer P2（10μM）	2μl
USA300 DNA	2μl
ddH_2O	19μl
Total	50μl

Amplification conditions：94℃ 5min 30Cycle（94℃ 30sec 、55℃ 30sec、72℃3min） 10℃ hold on

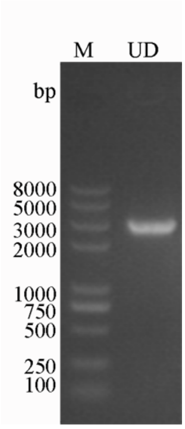

Figure 1: Electrophoresis detection of *clpB* gene amplification

Preparation of pCM carriers

Using the laboratory-preserved pCM plasmid as the template, the vector was reverse-amplified with the primers pCM-his-F/pCM-*clpB*-R

System grouping	Reaction volume
2×superpfu PCR mix	25μl
Primer P1（10μM）	2μl
Primer P2（10μM）	2μl
Template	1μl
ddH_2O	20μl
Total	50μl

Amplification conditions：94℃ 5min 30Cycle（94℃ 30sec 、55℃ 30sec、72℃3min） 10℃ hold on.

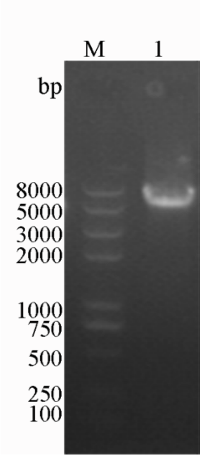

Figure 2: pCM carrier reverse-expanded 1% agarose gel electrophoresis detection

Seamless clone connection

Amplified *clpB* gene fragments and pCM vector fragments

System grouping	Reaction volume
5×infusion cloing mix	2μl
clpB recovery fragment	6μl
Recovery fragment of pCM anti-expansion carrier	2μl
Total	10μl

After thorough mixing, place on ice for 30 minutes, transform Ecoli DH5alphA competent cells, spread Amp plates, and incubate at 37 ° C. The clones grown were identified by colony PCR.

Identification primer: *clpB*-CX-F/M13-R

System: Select the monoclonal cells and mix them evenly in 10μl of sterile water. Take 0.5μl as the template and follow the system as follows:

System grouping	Reaction volume
2×Taq PCR mix	5μl
clpB-CX-F（10μM）	0.5μl
M13-R（10μM）	0.5μl
Bacterial liquid	0.5μl
ddH_2O	2.5μl
Total	10μl

Amplification conditions：94℃ 5min，30cycle（94℃ 30sec，55℃ 30sec ，72℃ 60sec），10℃ hold on.

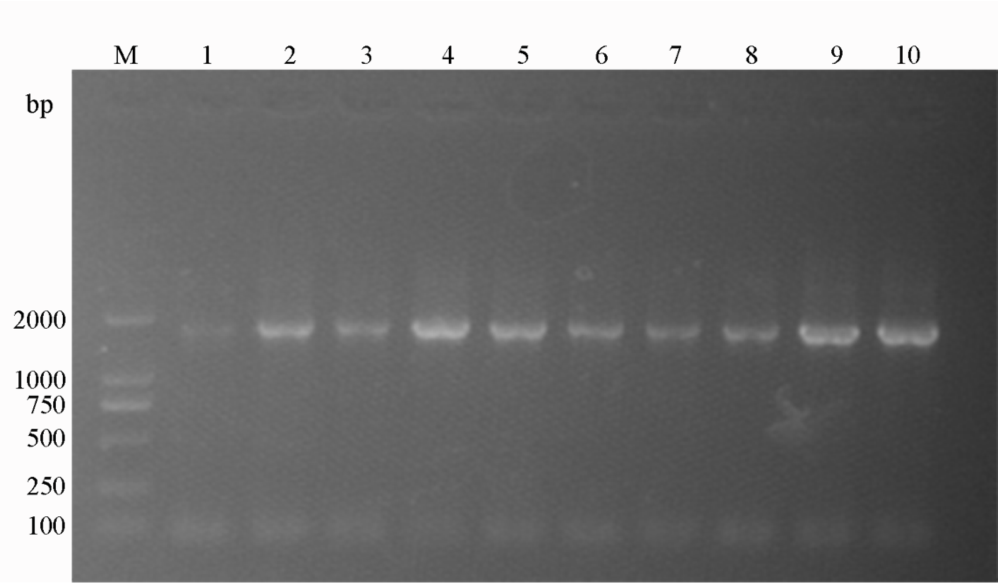

Figure 3: PCR identification of Ecoli DH5alphA colonies transformed from *clpB*-pCM

Note: Colonies 1 to 10 in the figure are randomly selected clones

Transformation screening of supplementary strains

Plasmid transformation RN4220

The correctly sequenced resupplement plasmids were added to the prepared RN4220 competent cells, placed on ice for 5 minutes, and then subjected to 2300V shock. After adding 1ml of TSB medium and restoring the culture for 1 hour, TSA (Cm 10μg/ml) plates were coated and the clones grown at 37 ° C were identified by colony PCR

Identification primer: *clpB*-CX-F/M13-R

System: Select the monoclonal cells and mix them evenly in 10μl of sterile water. Take 0.5μl as the template and follow the system as follows:

System grouping	Reaction volume
KOD one	5μl
clpB-CX-F（10μM）	0.5μl
M13-R（10μM）	0.5μl
Bacterial liquid	0.5μl
ddH_2O	2.5μl
Total	10μl

Amplification conditions：94℃ 5min, 32cycle（98℃ 5sec, 55℃ 5sec ，68℃ 30sec）, 10℃ hold on.

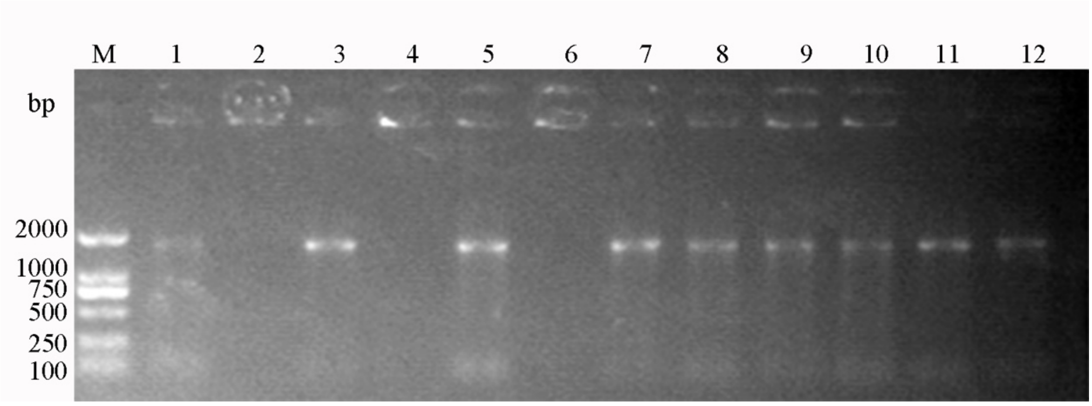

Figure 4: PCR identification of RN4220 colonies transformed from *clpB*-pCM

USA300 ∆*clpB* phage transduction

RN4220*clpB*-pCM phage was prepared and USA300 ∆*clpB* was transformed by phage transduction method

Coat TSA (Cm 10μg/ml) plates and incubate at 37 °C

Colony PCR identification

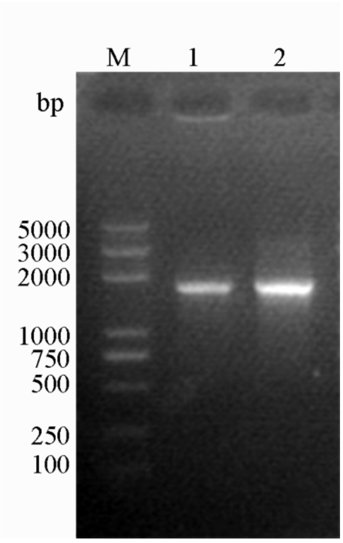

Figure 5 PCR identification of *clpB*-pCM USA300 ∆*clpB* transduction colonies

Positive clones were inoculated into 5ml of TSB (Cm10μg/ml) and incubated overnight at 37 °C. The preserved strains were the constructed replacement strains.

Concatenated sequence：
TGCTCAATTTTTGTTTGTAGAATTAGAATATATTTATTTGGCTCATATTTGCTTTTTAAAAGCTTGC
ATGCCTGCAGGTCGACTCTAGAGGATCCGCTAGCCTGATATTTTTGACTAAACCAAATGCTAAC
CCAGAAATACAATCACTGTGTCCATATGTTTAATAAGTCTAGTTAATGTGTAACGTAACATTAGC
TAGATTTTTTTATTCAAAAAAATATTTACAAATATTAGGAAATTTAAGTGTAAAAGAGTTGATAAA
TGATTATATTGGGACTATAATATAATTAAGGTCAAAGAAAGTCAAAGTCAAAAGTGCAACTTGA
CCAACTCTACAAATTAGAGGTGAAATTATATGGATATAAATAAAATGACATATGCTGTTCAAAGT
GCTTTACAACAAGCAGTTGAACTGAGTCAGCAACATAAATTACAAAATATAGAAATTGAGGCAA
TTTTAAGCGCTGCCTTAAATGAAAGTGAAAGCTTATATAAAAGTATTTTAGAACGAGCAAATATT
GAGGTAGATCAATTAAACAAAGCTTATGAAGACAAACTAAACACGTATGCATCTGTAGAAGGTG
ACAATATACAATATGGTCAATATATTAGCCAACAAGCAAACCAATTGATAACTAAGGCTGAATC
ATACATGAAAGAATATGAAGATGAATATATTTCAATGGAGCATATTTTACGTTCGGCAATGGACA
TTGATCAAACAACAAAACATTATATAAATAATAAAGTAGAAGTTATCAAAGAAATTATTAAAAAA
GTAAGAGGGGGAAATCACGTGACATCACAAAATCCAGAAGTTAATTACGAAGCATTAGCTAAA
TATGGCCGCGACTTAGTAGAAGAAGTTAGACAAGGTAAAATGGATCCTGTTATAGGAAGAGAT
GAAGAAATTCGAAATACGATTCGTATTTTAAGTCGTAAAACTAAAAACAACCCTGTGCTCATTGG
TGAACCAGGTGTTGGTAAAACTGCAATTGTTGAAGGATTAGCGCAACGTATAGTTAAGAAAGAT
GTGCCAGAATCATTATTAGATAAAACTGTTTTTGAGTTAGATTTAAGCGCATTAGTAGCGGGCGC
TAAATATCGTGGTGAATTTGAAGAGAGATTAAAAGCAGTCCTAAAAGAAGTTAAAGAGTCTGAT
GGTAGAATTATATTATTTATTGATGAAATCCATATGCTTGTAGGTGCTGGTAAAACAGATGGTGC
CATGGATGCAGGCAACATGCTAAAACCAATGTTAGCACGAGGAGAGTTACATTGTATTGGTGCA
ACAACTTTAAATGAATATCGAGAATATATTGAAAAAGATTCGGCATTAGAGCGTCGTTTCCAAAA
AGTAGCAGTTAGTGAGCCTGATGTTGAAGATACAATTTCAATTTTACGTGGTTTAAAAGAACGAT
ATGAAGTGTATCATGGTGTGCGTATTCAAGATAGAGCCTTAGTTGCTGCCGCTGAATTGTCTGAT
CGTTACATCACTGATCGTTTTTTACCAGATAAAGCGATTGATTTAGTTGACCAAGCATGTGCAAC
AATTCGTACGGAAATGGGATCAAATCCAACTGAATTGGATCAAGTTAATAGACGTGTCATGCAA
TTAGAAATTGAAGAAAGCGCACTTAAAAATGAATCTGACAATGCGAGCAAACAGAGATTACAA
GAACTACAAGAAGAGCTTGCCAATGAAAAAGAGAAACAAGCAGCACTTCAATCTCGTGTAGAA
TCAGAAAAAGAAAAAATAGCAAATTTACAAGAAAAACGTGCGCAACTAGATGAAAGTAGACAA
GCGTTGGAAGATGCACAAACAAATAACAATTTAGAAAAAGCTGCTGAACTACAATATGGAACA
ATTCCTCAATTGGAAAAAGAACTTAGAGAATTAGAGGATAATTTCCAAGATGAGCAAGGTGAAG
ATACAGATCGAATGATTCGTGAAGTTGTAACAGACGAAGAAATTGGCGATATTGTCAGCCAATG
GACAGGCATACCAGTTTCAAAATTAGTTGAAACAGAACGTGAAAAATTACTTCACTTAAGTGAC
ATCTTGCATAAACGTGTTGTAGGTCAAGATAAAGCGGTTGACCTGGTTTCAGATGCAGTAGTTA
GAGCAAGAGCAGGTATTAAAGATCCAAACAGACCTATTGGTAGTTTCTTATTCCTAGGTCCAACT
GGAGTAGGTAAAACTGAATTAGCTAAATCATTAGCTGCATCATTATTTGATTCTGAAAAACATAT
GATTCGTATTGATATGAGTGAATATATGGAAAAACATGCAGTATCAAGATTGATAGGGGCACCT
CCAGGATATATTGGACATGATGAAGGGGGTCAATTAACTGAAGCGGTTCGTCGTAATCCATACT
CAGTTATTTTATTAGATGAGGTTGAAAAAGCGCATACTGACGTCTTTAATGTATTATTGCAAATTT
TAGATGAAGGCCGTTTAACTGATTCTAAAGGACGTAGCGTTGATTTTAAAAATACTATTATTATTA
TGACAAGTAATATTGGATCTCAAGTTTTATTAGAAAACGTAAAAGAGACTGGTGAAATTACAGA
ATCAACAGAAAAAGCTGTTATGACAAGTTTAAATGCATATTTCAAACCAGAAATTTTGAATCGTA
TGGATGATATCGTATTATTTAAACCATTATCTATTGATGACATGAGTATGATTGTAGATAAAATCT

TAACGCAATTAAATATAAGATTATTAGAACAACGAATCTCAATTGAAGTTTCTGATGATGCTAAA
GCTTGGCTAGGTCAAGAAGCTTATGAACCTCAATACGGTGCAAGACCATTAAAACGTTTTGTAC
AACGCCAAATTGAAACACCATTAGCACGTATGATGATTAAAGAGGGATTCCCAGAAGGTACAA
CGATTAAAGTTAATTTAAATTCAGACAATAACTTAACGTTTAATGTTGAAAAAATTCATGAACAC
CACCACCACCACCACCACTAAGAATTCGTAATCATGTCATAGCTGTTTCCTGTGTGAAATGTTAT
CCGCTCACAATTCCACACAA

Expected comparison：

```
Fast alignment of DNA sequences DNAMAN2 and DNAMAN1

Ktuple=2  Gap_penalty=7

Upper line: DNAMAN2, from 1 to 2851
Lower line: DNAMAN1, from 136 to 2986

DNAMAN2:DNAMAN1 identity= 100.00%(2852/2852) gap=0.00%(0/2852)
1    AAATACAATCACTGTGTCCATATGTTTAATAAGTCTAGTTAATGTGTAACGTAACATTAG
     ||||||||||||||||||||||||||||||||||||||||||||||||||||||||||||
136  AAATACAATCACTGTGTCCATATGTTTAATAAGTCTAGTTAATGTGTAACGTAACATTAG

61   CTAGATTTTTTTATTCAAAAAAATATTTACAAATATTAGGAAATTTAAGTGTAAAAGAGT
     ||||||||||||||||||||||||||||||||||||||||||||||||||||||||||||
196  CTAGATTTTTTTATTCAAAAAAATATTTACAAATATTAGGAAATTTAAGTGTAAAAGAGT

121  TGATAAATGATTATATTGGGACTATAATATAATTAAGGTCAAAGAAAGTCAAAGTCAAAA
     ||||||||||||||||||||||||||||||||||||||||||||||||||||||||||||
256  TGATAAATGATTATATTGGGACTATAATATAATTAAGGTCAAAGAAAGTCAAAGTCAAAA

181  GTGCAACTTGACCAACTCTACAAATTAGAGGTGAAATTATATGGATATAAATAAAATGAC
     ||||||||||||||||||||||||||||||||||||||||||||||||||||||||||||
316  GTGCAACTTGACCAACTCTACAAATTAGAGGTGAAATTATATGGATATAAATAAAATGAC

241  ATATGCTGTTCAAAGTGCTTTACAACAAGCAGTTGAACTGAGTCAGCAACATAAATTACA
     ||||||||||||||||||||||||||||||||||||||||||||||||||||||||||||
376  ATATGCTGTTCAAAGTGCTTTACAACAAGCAGTTGAACTGAGTCAGCAACATAAATTACA

301  AAATATAGAAATTGAGGCAATTTTAAGCGCTGCCTTAAATGAAAGTGAAAGCTTATATAA
     ||||||||||||||||||||||||||||||||||||||||||||||||||||||||||||
436  AAATATAGAAATTGAGGCAATTTTAAGCGCTGCCTTAAATGAAAGTGAAAGCTTATATAA

361  AAGTATTTTAGAACGAGCAAATATTGAGGTAGATCAATTAAACAAAGCTTATGAAGACAA
     ||||||||||||||||||||||||||||||||||||||||||||||||||||||||||||
496  AAGTATTTTAGAACGAGCAAATATTGAGGTAGATCAATTAAACAAAGCTTATGAAGACAA

421  ACTAAACACGTATGCATCTGTAGAAGGTGACAATATACAATATGGTCAATATATTAGCCA
```

```
     ||||||||||||||||||||||||||||||||||||||||||||||||||||||||||||
556  ACTAAACACGTATGCATCTGTAGAAGGTGACAATATACAATATGGTCAATATATTAGCCA

481  ACAAGCAAACCAATTGATAACTAAGGCTGAATCATACATGAAAGAATATGAAGATGAATA
     ||||||||||||||||||||||||||||||||||||||||||||||||||||||||||||
616  ACAAGCAAACCAATTGATAACTAAGGCTGAATCATACATGAAAGAATATGAAGATGAATA

541  TATTTCAATGGAGCATATTTTACGTTCGGCAATGGACATTGATCAAACAACAAAACATTA
     ||||||||||||||||||||||||||||||||||||||||||||||||||||||||||||
676  TATTTCAATGGAGCATATTTTACGTTCGGCAATGGACATTGATCAAACAACAAAACATTA

601  TATAAATAATAAAGTAGAAGTTATCAAAGAAATTATTAAAAAAGTAAGAGGGGGAAATCA
     ||||||||||||||||||||||||||||||||||||||||||||||||||||||||||||
736  TATAAATAATAAAGTAGAAGTTATCAAAGAAATTATTAAAAAAGTAAGAGGGGGAAATCA

661  CGTGACATCACAAAATCCAGAAGTTAATTACGAAGCATTAGCTAAATATGGCCGCGACTT
     ||||||||||||||||||||||||||||||||||||||||||||||||||||||||||||
796  CGTGACATCACAAAATCCAGAAGTTAATTACGAAGCATTAGCTAAATATGGCCGCGACTT

721  AGTAGAAGAAGTTAGACAAGGTAAAATGGATCCTGTTATAGGAAGAGATGAAGAAATTCG
     ||||||||||||||||||||||||||||||||||||||||||||||||||||||||||||
856  AGTAGAAGAAGTTAGACAAGGTAAAATGGATCCTGTTATAGGAAGAGATGAAGAAATTCG

781  AAATACGATTCGTATTTTAAGTCGTAAAACTAAAAACAACCCTGTGCTCATTGGTGAACC
     ||||||||||||||||||||||||||||||||||||||||||||||||||||||||||||
916  AAATACGATTCGTATTTTAAGTCGTAAAACTAAAAACAACCCTGTGCTCATTGGTGAACC

841  AGGTGTTGGTAAAACTGCAATTGTTGAAGGATTAGCGCAACGTATAGTTAAGAAAGATGT
     ||||||||||||||||||||||||||||||||||||||||||||||||||||||||||||
976  AGGTGTTGGTAAAACTGCAATTGTTGAAGGATTAGCGCAACGTATAGTTAAGAAAGATGT

901  GCCAGAATCATTATTAGATAAAACTGTTTTTGAGTTAGATTTAAGCGCATTAGTAGCGGG
     ||||||||||||||||||||||||||||||||||||||||||||||||||||||||||||
1036 GCCAGAATCATTATTAGATAAAACTGTTTTTGAGTTAGATTTAAGCGCATTAGTAGCGGG

961  CGCTAAATATCGTGGTGAATTTGAAGAGAGATTAAAAGCAGTCCTAAAAGAAGTTAAAGA
     ||||||||||||||||||||||||||||||||||||||||||||||||||||||||||||
1096 CGCTAAATATCGTGGTGAATTTGAAGAGAGATTAAAAGCAGTCCTAAAAGAAGTTAAAGA

1021 GTCTGATGGTAGAATTATATTATTTATTGATGAAATCCATATGCTTGTAGGTGCTGGTAA
     ||||||||||||||||||||||||||||||||||||||||||||||||||||||||||||
1156 GTCTGATGGTAGAATTATATTATTTATTGATGAAATCCATATGCTTGTAGGTGCTGGTAA

1081 AACAGATGGTGCCATGGATGCAGGCAACATGCTAAAACCAATGTTAGCACGAGGAGAGTT
```

```
     ||||||||||||||||||||||||||||||||||||||||||||||||||||||||||||
1216 AACAGATGGTGCCATGGATGCAGGCAACATGCTAAAACCAATGTTAGCACGAGGAGAGTT

1141 ACATTGTATTGGTGCAACAACTTTAAATGAATATCGAGAATATATTGAAAAAGATTCGGC
     ||||||||||||||||||||||||||||||||||||||||||||||||||||||||||||
1276 ACATTGTATTGGTGCAACAACTTTAAATGAATATCGAGAATATATTGAAAAAGATTCGGC

1201 ATTAGAGCGTCGTTTCCAAAAAGTAGCAGTTAGTGAGCCTGATGTTGAAGATACAATTTC
     ||||||||||||||||||||||||||||||||||||||||||||||||||||||||||||
1336 ATTAGAGCGTCGTTTCCAAAAAGTAGCAGTTAGTGAGCCTGATGTTGAAGATACAATTTC

1261 AATTTTACGTGGTTTAAAAGAACGATATGAAGTGTATCATGGTGTGCGTATTCAAGATAG
     ||||||||||||||||||||||||||||||||||||||||||||||||||||||||||||
1396 AATTTTACGTGGTTTAAAAGAACGATATGAAGTGTATCATGGTGTGCGTATTCAAGATAG

1321 AGCCTTAGTTGCTGCCGCTGAATTGTCTGATCGTTACATCACTGATCGTTTTTTACCAGA
     ||||||||||||||||||||||||||||||||||||||||||||||||||||||||||||
1456 AGCCTTAGTTGCTGCCGCTGAATTGTCTGATCGTTACATCACTGATCGTTTTTTACCAGA

1381 TAAAGCGATTGATTTAGTTGACCAAGCATGTGCAACAATTCGTACGGAAATGGGATCAAA
     ||||||||||||||||||||||||||||||||||||||||||||||||||||||||||||
1516 TAAAGCGATTGATTTAGTTGACCAAGCATGTGCAACAATTCGTACGGAAATGGGATCAAA

1441 TCCAACTGAATTGGATCAAGTTAATAGACGTGTCATGCAATTAGAAATTGAAGAAAGCGC
     ||||||||||||||||||||||||||||||||||||||||||||||||||||||||||||
1576 TCCAACTGAATTGGATCAAGTTAATAGACGTGTCATGCAATTAGAAATTGAAGAAAGCGC

1501 ACTTAAAAATGAATCTGACAATGCGAGCAAACAGAGATTACAAGAACTACAAGAAGAGCT
     ||||||||||||||||||||||||||||||||||||||||||||||||||||||||||||
1636 ACTTAAAAATGAATCTGACAATGCGAGCAAACAGAGATTACAAGAACTACAAGAAGAGCT

1561 TGCCAATGAAAAAGAGAAACAAGCAGCACTTCAATCTCGTGTAGAATCAGAAAAAGAAAA
     ||||||||||||||||||||||||||||||||||||||||||||||||||||||||||||
1696 TGCCAATGAAAAAGAGAAACAAGCAGCACTTCAATCTCGTGTAGAATCAGAAAAAGAAAA

1621 AATAGCAAATTTACAAGAAAAACGTGCGCAACTAGATGAAAGTAGACAAGCGTTGGAAGA
     ||||||||||||||||||||||||||||||||||||||||||||||||||||||||||||
1756 AATAGCAAATTTACAAGAAAAACGTGCGCAACTAGATGAAAGTAGACAAGCGTTGGAAGA

1681 TGCACAAACAAATAACAATTTAGAAAAAGCTGCTGAACTACAATATGGAACAATTCCTCA
     ||||||||||||||||||||||||||||||||||||||||||||||||||||||||||||
1816 TGCACAAACAAATAACAATTTAGAAAAAGCTGCTGAACTACAATATGGAACAATTCCTCA

1741 ATTGGAAAAAGAACTTAGAGAATTAGAGGATAATTTCCAAGATGAGCAAGGTGAAGATAC
```

```
     ||||||||||||||||||||||||||||||||||||||||||||||||||||||||||||
1876 ATTGGAAAAAGAACTTAGAGAATTAGAGGATAATTTCCAAGATGAGCAAGGTGAAGATAC

1801 AGATCGAATGATTCGTGAAGTTGTAACAGACGAAGAAATTGGCGATATTGTCAGCCAATG
     ||||||||||||||||||||||||||||||||||||||||||||||||||||||||||||
1936 AGATCGAATGATTCGTGAAGTTGTAACAGACGAAGAAATTGGCGATATTGTCAGCCAATG

1861 GACAGGCATACCAGTTTCAAAATTAGTTGAAACAGAACGTGAAAAATTACTTCACTTAAG
     ||||||||||||||||||||||||||||||||||||||||||||||||||||||||||||
1996 GACAGGCATACCAGTTTCAAAATTAGTTGAAACAGAACGTGAAAAATTACTTCACTTAAG

1921 TGACATCTTGCATAAACGTGTTGTAGGTCAAGATAAAGCGGTTGACCTGGTTTCAGATGC
     ||||||||||||||||||||||||||||||||||||||||||||||||||||||||||||
2056 TGACATCTTGCATAAACGTGTTGTAGGTCAAGATAAAGCGGTTGACCTGGTTTCAGATGC

1981 AGTAGTTAGAGCAAGAGCAGGTATTAAAGATCCAAACAGACCTATTGGTAGTTTCTTATT
     ||||||||||||||||||||||||||||||||||||||||||||||||||||||||||||
2116 AGTAGTTAGAGCAAGAGCAGGTATTAAAGATCCAAACAGACCTATTGGTAGTTTCTTATT

2041 CCTAGGTCCAACTGGAGTAGGTAAAACTGAATTAGCTAAATCATTAGCTGCATCATTATT
     ||||||||||||||||||||||||||||||||||||||||||||||||||||||||||||
2176 CCTAGGTCCAACTGGAGTAGGTAAAACTGAATTAGCTAAATCATTAGCTGCATCATTATT

2101 TGATTCTGAAAAACATATGATTCGTATTGATATGAGTGAATATATGGAAAAACATGCAGT
     ||||||||||||||||||||||||||||||||||||||||||||||||||||||||||||
2236 TGATTCTGAAAAACATATGATTCGTATTGATATGAGTGAATATATGGAAAAACATGCAGT

2161 ATCAAGATTGATAGGGGCACCTCCAGGATATATTGGACATGATGAAGGGGGTCAATTAAC
     ||||||||||||||||||||||||||||||||||||||||||||||||||||||||||||
2296 ATCAAGATTGATAGGGGCACCTCCAGGATATATTGGACATGATGAAGGGGGTCAATTAAC

2221 TGAAGCGGTTCGTCGTAATCCATACTCAGTTATTTTATTAGATGAGGTTGAAAAAGCGCA
     ||||||||||||||||||||||||||||||||||||||||||||||||||||||||||||
2356 TGAAGCGGTTCGTCGTAATCCATACTCAGTTATTTTATTAGATGAGGTTGAAAAAGCGCA

2281 TACTGACGTCTTTAATGTATTATTGCAAATTTTAGATGAAGGCCGTTTAACTGATTCTAA
     ||||||||||||||||||||||||||||||||||||||||||||||||||||||||||||
2416 TACTGACGTCTTTAATGTATTATTGCAAATTTTAGATGAAGGCCGTTTAACTGATTCTAA

2341 AGGACGTAGCGTTGATTTTAAAAATACTATTATTATTATGACAAGTAATATTGGATCTCA
     ||||||||||||||||||||||||||||||||||||||||||||||||||||||||||||
2476 AGGACGTAGCGTTGATTTTAAAAATACTATTATTATTATGACAAGTAATATTGGATCTCA

2401 AGTTTTATTAGAAAACGTAAAAGAGACTGGTGAAATTACAGAATCAACAGAAAAAGCTGT
```

```
     ||||||||||||||||||||||||||||||||||||||||||||||||||||||||||||
2536 AGTTTTATTAGAAAACGTAAAAGAGACTGGTGAAATTACAGAATCAACAGAAAAAGCTGT

2461 TATGACAAGTTTAAATGCATATTTCAAACCAGAAATTTTGAATCGTATGGATGATATCGT
     ||||||||||||||||||||||||||||||||||||||||||||||||||||||||||||
2596 TATGACAAGTTTAAATGCATATTTCAAACCAGAAATTTTGAATCGTATGGATGATATCGT

2521 ATTATTTAAACCATTATCTATTGATGACATGAGTATGATTGTAGATAAAATCTTAACGCA
     ||||||||||||||||||||||||||||||||||||||||||||||||||||||||||||
2656 ATTATTTAAACCATTATCTATTGATGACATGAGTATGATTGTAGATAAAATCTTAACGCA

2581 ATTAAATATAAGATTATTAGAACAACGAATCTCAATTGAAGTTTCTGATGATGCTAAAGC
     ||||||||||||||||||||||||||||||||||||||||||||||||||||||||||||
2716 ATTAAATATAAGATTATTAGAACAACGAATCTCAATTGAAGTTTCTGATGATGCTAAAGC

2641 TTGGCTAGGTCAAGAAGCTTATGAACCTCAATACGGTGCAAGACCATTAAAACGTTTTGT
     ||||||||||||||||||||||||||||||||||||||||||||||||||||||||||||
2776 TTGGCTAGGTCAAGAAGCTTATGAACCTCAATACGGTGCAAGACCATTAAAACGTTTTGT

2701 ACAACGCCAAATTGAAACACCATTAGCACGTATGATGATTAAAGAGGGATTCCCAGAAGG
     ||||||||||||||||||||||||||||||||||||||||||||||||||||||||||||
2836 ACAACGCCAAATTGAAACACCATTAGCACGTATGATGATTAAAGAGGGATTCCCAGAAGG

2761 TACAACGATTAAAGTTAATTTAAATTCAGACAATAACTTAACGTTTAATGTTGAAAAAAT
     ||||||||||||||||||||||||||||||||||||||||||||||||||||||||||||
2896 TACAACGATTAAAGTTAATTTAAATTCAGACAATAACTTAACGTTTAATGTTGAAAAAAT

2821 TCATGAACACCACCACCACCACCACCACTAAG
     ||||||||||||||||||||||||||||||||
2956 TCATGAACACCACCACCACCACCACCACTAAGAATTCGTAATCATGTCATAGCTGTTTCC
```
