# Supplementary material for: ClpB affects biofilm formation in methicillin-resistant Staphylococcus aureus
Source: Front Microbiol. 2025 Dec 4;16:1723924. doi: 10.3389/fmicb.2025.1723924 (PMC12711867; doi:10.3389/fmicb.2025.1723924)
Supplement: Supplementary file 2 [file Data_Sheet_2.docx]

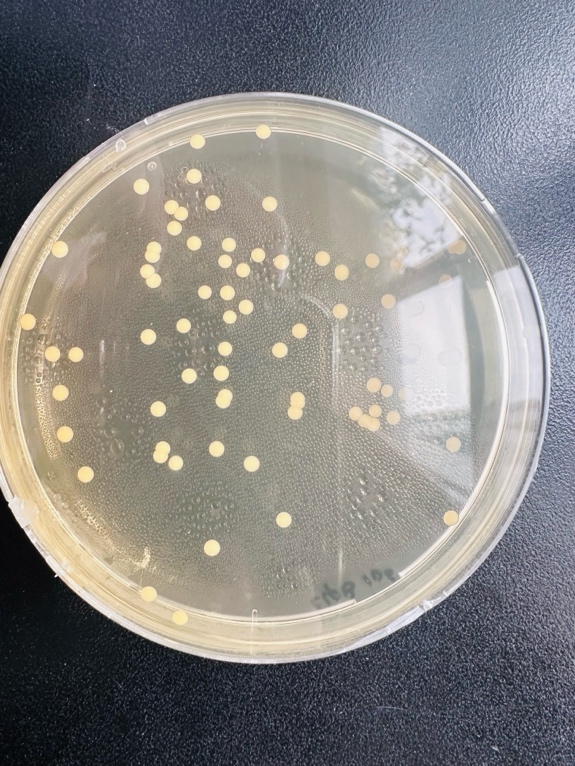

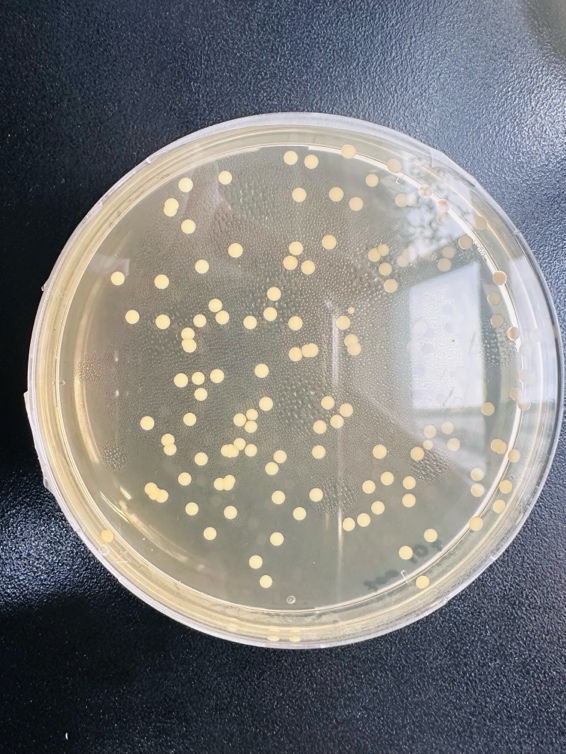


Figure S2: Count of colonies of wild and Δ*clpB* mutant strains. On the left is a Δ*clpB* mutant and on the right is a wild strain at a dilution of 10^6^.

The dispersion effect of Staphylococcus aureus biofilm was evaluated by plate counting method. After strain culture, bacterial suspension was diluted (10⁻³~10⁻^7^), and the dilution of 10⁻^6^ (the number of plate colonies was in the range of 30~300) was selected for counting.

Formula: CFU/mL= Colony count* Dilution ratio/ Inoculum size

Result:

Δ*clpB*: CFU/mL=75*10^6^/0.1=7.5*10^8^ CFU/mL(n=3)

WT: CFU/mL=135*10^6^/0.1=1.35*10^9^ CFU/mL(n=3)
